# CREG1 deficiency impaired myoblast differentiation and skeletal muscle regeneration

**DOI:** 10.1002/jcsm.13427

**Published:** 2024-01-25

**Authors:** Haixu Song, Xiaoxiang Tian, Lianqi He, Dan Liu, Jiayin Li, Zhu Mei, Ting Zhou, Chunying Liu, Jiaqi He, Xiaodong Jia, Zheming Yang, Chenghui Yan, Yaling Han

**Affiliations:** ^1^ Department of Cardiology, Cardiovascular Research Institute, State Key Laboratory of Frigid Zone Cardiovascular Disease General Hospital of Northern Theater Command Shenyang China

**Keywords:** AMPKa1, C‐CBL, CREG1, Satellite cells, Skeletal muscle regeneration

## Abstract

**Background:**

CREG1 (cellular repressor of E1A‐stimulated genes 1) is a protein involved in cellular differentiation and homeostasis regulation. However, its role in skeletal muscle satellite cells differentiation and muscle regeneration is poorly understood. This study aimed to investigate the role of CREG1 in myogenesis and muscle regeneration.

**Methods:**

RNA sequencing data (GSE8479) was analysed from the Gene Expression Omnibus database (GEO, https://www.ncbi.nlm.nih.gov/geo/query/acc.cgi). We generated *Creg1* knockdown and skeletal muscle satellite cells specific *Creg1* overexpression mice mediated by adeno‐associated virus serotype 9 (AAV9), skeletal muscle mature myofibre *Creg1* knockout mice (myoblast/*Creg1MKO*), and control mice *Creg1* ^*flox/flox*^ (*Creg1* ^*fl/fl*^) as in vivo models. The mice were injected into tibialis anterior (TA) muscle with 100 μL of 10 μM cardiotoxin to establish a muscle regeneration model. *Creg1* ^*fl/fl*^ and *Creg1MKO* mice were treated with AAV‐sh‐C‐Cbl (2 × 10^10^ genomic copies/mouse) to silence *C‐Cbl* in the TA muscle. 293T and C2C12 cells were transfected with plasmids using lipofectamine RNAi MAX in vitro. Mass spectrometry analyses and RNA sequencing transcriptomic assay were performed.

**Results:**

We analysed the transcriptional profiles of the skeletal muscle biopsies from healthy older (*N* = 25) and younger (*N* = 26) adult men and women in GSE8479 database, and the results showed that *Creg1* was associated with human sarcopenia. We found that *Creg1* knockdown mice regenerated less newly formed fibres in response to cardiotoxin injection (~30% reduction, *P* < 0.01); however, muscle satellite cells specific *Creg1* overexpression mice regenerated more newly formed fibres (~20% increase, *P* < 0.05). AMPKa1 is known as a key mediator in the muscle regeneration process. Our results revealed that CREG1 deficiency inhibited AMPKa1 signalling through C‐CBL E3‐ubiquitin ligase‐mediated AMPKa1 degradation (*P* < 0.01). C‐CBL‐mediated AMPKa1 ubiquitination was attributed to the K48‐linked polyubiquitination of AMPKa1 at K396 and that the modification played an important role in the regulation of AMPKa1 protein stability. We also found that *Creg1MKO* mice regenerated less newly formed fibres compared with *Creg1* ^*fl/fl*^ mice (~30% reduction, *P* < 0.01). RNA‐seq analysis showed that CREG1 deletion in impaired muscles led to the upregulation of inflammation and DKK3 expression. The TA muscles of *Creg1MKO* mice were injected with AAV‐vector or AAV‐shC‐Cbl, silencing C‐CBL (*P* < 0.01) in the skeletal muscles of *Creg1MKO* mice significantly improved muscle regeneration induced by CTX injury (*P* < 0.01).

**Conclusions:**

Our findings suggest that CREG1 may be a potential therapeutic target for skeletal muscle regeneration.

## Introduction

Skeletal muscle, as the largest tissue in mammals accounts for approximately 40% of the body weight and is responsible for the support, movement, and homeostasis of organisms.^1^ Muscle regeneration is an essential physiological process in the skeletal muscles. In both muscle trauma and exercise‐induced muscle damage, skeletal muscle regeneration is necessary for recovery after injury.^2^, ^3^, ^4^ Myofibre necrosis and muscle mass loss are common in injured muscle, leading to muscle malfunctions. Fortunately, the skeletal muscles of adults have an extraordinary capacity to repair these injuries. This capacity for regeneration is due to the presence of muscle‐resident stem cells, also called satellite cells, which are located between the sarcolemma and basal lamina.^5^, ^6^

In response to injury or disease, satellite cells can be activated to generate myoblasts that proliferate and differentiate into multinucleated myotubes.^7^ Myoblast proliferation and differentiation are called myogenesis, which is not only important for muscle growth and development but is also necessary for muscle regeneration. The damaged muscle fibres are fused with myogenic cells for repair or to generate new muscle fibres to replace the necrotic fibres. A decline in the muscle regeneration capacity often leads to muscle atrophy and contractile function impairment.^8^, ^9^ Successful muscle regeneration requires both the right number and appropriate myogenic differentiation of satellite cells. Previous studies have demonstrated that AMPK (5′‐adenosine monophosphate‐activated protein kinase) plays a critical role in regulating muscle regeneration and growth, especially with AMPKα1, the dominant AMPKα isoform in satellite cells, playing the prominent role in facilitating muscle regeneration.^10^, ^11^ Although numerous studies have attempted to clarify the complicated muscle regeneration process, the explicit mechanisms of muscle regeneration remain unclear. Besides the skeletal muscle satellite cells, mature myofibre as an important endocrine organ, could produce and release myokines through autocrine and paracrine ways to regulate skeletal muscle homeostasis, including modulating muscle satellite cells differentiation and involving in muscle atrophy.^12^ However, the role of mature myofibre in regulating muscle stem cell regenerative capacity remains further explored.

CREG1 (cellular repressor of E1A‐stimulated genes 1) is a protein involved in cellular differentiation and regulation of homeostasis. Previous studies have shown that CREG1 is highly expressed in differentiated and mature tissues and cells, such as differentiated smooth muscle cells and mature cardiomyocytes.^13^, ^14^ It has been demonstrated that CREG1 is a senescence‐related protein, and its expression is reduced with age owing to high methylation in its gene promoter region.^15^ A recent study showed that CREG1 is expressed in the skeletal muscle and plays an important role in maintaining exercise capacity in adult mice.^16^ However, its role in myogenesis and muscle regeneration remains uninvestigated.

In this study, we aimed to investigate the role of CREG1 in skeletal muscle regeneration. We revealed that the expression of CREG1 mRNA and protein levels were significantly upregulated in skeletal muscle regeneration. Using *Creg1* knockdown and skeletal muscle satellite cell specific *Creg1* overexpression mice mediated by adeno‐associated virus serotype 9 (AAV9), skeletal muscle‐specific *Creg1* knockout mice (myoblast/*Creg1MKO*) and *Creg1* ^*flox/flox*^ (*Creg1* ^*fl/fl*^) as in vivo models, and analysed their regeneration capacity. We uncover the important role of CREG1 regulating muscle satellite cells differentiation and muscle regeneration. CREG1 may be a potential therapeutic target of muscle regeneration impairment and atrophy.

## Methods

### Experimental animals

*Creg1* knockdown and skeletal muscle satellite cell specific *Creg1* overexpression mice mediated by adeno‐associated virus serotype 9 (AAV9). Skeletal muscle‐specific *Creg1* knockout mice (myoblast/*Creg1MKO*) and control mice in the same litter (*Creg1* ^*fl/fl*^) were generated by Gempharmatech Co., Ltd (Shanghai, China). All male mice were 8‐week‐old and used in a C57BL/6 background. All mice were fed and placed in a 12:12 h light/dark cycle system using an automated light‐switching project and temperature‐controlled conditions at 22°C. The mice were injected into each tibialis anterior (TA) muscle with 100 μL of 10 μM cardiotoxin (CTX, Sigma, USA) in order to generate a muscle regeneration model. Animals were sacrificed at 3, 7, and 14 days after injection for histological analysis. *Creg1* ^*fl/fl*^ and *Creg1MKO* mice were treated with AAV‐sh‐C‐Cbl (BOIO‐HYKY‐220810027, 2 × 10^10^ genomic copies/mouse) to silence *C‐Cbl* in the TA muscle. All experiments were approved by the Animal Ethics Committee of Shenyang General Hospital and conducted in accordance with the existing guidelines on the care and use of laboratory animals.

### Construction of the adeno‐associated virus

AAV9‐EGFP‐U6‐shCreg1(AAV‐shCreg1) and AAV9‐MHCK7‐mCreg1(AAV9‐Creg1) were constructed by YUNZHOU Biosciences (Guangzhou, China), shCreg1: GCCACTATCTCCACAATAA. Mice were intravenously injected with AAV‐vector, AAV‐shCreg1 and AAV‐Creg1 (4 × 10^11^ genome copies per mouse in 200 μL of PBS), to achieve CREG1 knockdown and overexpression. *Creg1* ^*fl/fl*^ and *Creg1MKO* mice were treated with AAV‐sh‐C‐Cbl (BOIO‐HYKY‐220810027, 2 × 10^10^ genomic copies/mouse) to silence *C‐Cbl* in the TA muscle, sh‐C‐Cbl: ACCAACTCCTCAAGATCATAT.

### Cell lines, adenovirus vector, plasmid, small interfering RNA, and transfection reagent

293T and C2C12 cell lines were obtained from the FuHeng Cell Center (Shanghai, China) and cultured in 5% CO_2_ at 37°C in 10% FBS (Gibco, Thermo Fisher Scientific) DMEM (Life Technologies Corporation, 2230808). When reaching 70–80% confluence, 293T and C2C12 cells were transfected with plasmids using lipofectamine RNAi MAX (Thermo Fisher Scientific, 2103411) following the manufacturer's instructions. C2C12 cells were transfected with the indicated adenovirus or siRNA (si*Ampka1*, si*C‐Cbl*, si*Itch*, and si*Creg1*). After infection, C2C12 cells were cultured in differentiation medium (2% horse serum, Solarbio, S9050) for at least 4 days to induce their differentiation into myotubes. C2C12 cells were treated with MG132 (Sigma‐Aldrich, M8699; 10 μmol/L) 24 h. Plasmids and adenoviral vectors construction were in the supporting information.

### Western blot and antibodies

Cells and homogenized TA and gastrocnemius (GAS) tissues were lysed in ice‐cold RIPA buffer (Thermo Fisher Scientific, UJ289235) for 30 min and then centrifuged at 12 000× *g* for 10 min at 4°C. Equal amounts of samples were separated by SDS‐PAGE (at 120 V for 1 h), and then proteins were transferred onto a polyvinylidene difluoride membrane (Merck Millipore Ltd. R9KA84149). Membranes were blocked in 5% nonfat milk, then were incubated with antibodies (in the supporting information). After incubation, three washes with TBST, membranes were incubated the HRP‐conjugated secondary antibody (1:5000; Jackson ImmunoResearch, 150783) for 2 h at room temperature. After four washes with TBST, membranes were scanned with ECL in Amersham Imager680 (Tokyo, Japan).

### Immunoprecipitation

The TA muscle, 293T or C2C12 cells were homogenized and lysed with immunoprecipitation (IP) lysis buffer (including protease inhibitor cocktail) for 30 min on ice. The lysate was then centrifuged at 12 000× *g* for 15 min. The protein was incubated with FLAG (Solarbio, M2510) or ubiquitin‐conjugated beads (MBL, D058‐8) and rotated overnight at 4°C. Then, the beads were washed three times with IP lysis buffer. Western blot was performed as described previously.

### Real‐time PCR

As described previously,^16^ according to the Eastep® Super kit's instructions (Promega, 0000287896), RNA was reverse transcribed using the SuperScript™ III First‐Strand Kit (Thermo Fisher Scientific, 18080400). Real‐time PCR was performed on an ABI 7300 PCR System. Primers are listed in the supporting information.

### Haematoxylin and eosin and immunohistochemical staining

All mice were anaesthetised with isoflurane and sacrificed. Isolated TA muscles were fixed using 4% formaldehyde. The fixed tissues were dehydrated, embedded in paraffin, sectioned, stained with eosin, and dehydrated with alcohol. Immunohistochemical analysis was performed as previously described.^16^

### Immunofluorescent staining and EMH^+^ muscle fibres

C2C12 cells were fixed using 4% formaldehyde for 15–30 min, then treated with 0.1% Triton X‐100 (Sangon Biotech, T0694) for 10 min permeabilization. The samples were analysed using anti‐MyHC (Abcam, ab207926), and the cells nuclei were stained with DAPI. The differentiated myotube index was calculated as the percentage of the total image number covered by differentiated myotubes, and the measurement was performed using ImageJ software. TA muscle sections were prepared using anti‐Laminin (Abcam, ab11575) and anti‐embryonic myosin heavy chain (MYH3, Abcam, F1.652).^2^

### Statistical analysis

All data are expressed as mean ± standard error (SEM). All data were analysed using SPSS 13.0 (SPSS, Chicago, USA) and GraphPad Prism 8.0 statistical software. The Shapiro–Wilk test was used for normality and Levene's test was used for homogeneity of variance. When the data conformed to both normal distribution and homogeneity of variance, differences between the two groups were compared using paired or unpaired *t*‐tests, one‐way ANOVA, and repeated measures of variance. When the data do not conform to a normal distribution, the method is a non‐parametric test. *P* < 0.05 was statistically significant.

## Results

### CREG1 was associated with human sarcopenia and skeletal muscle regeneration

To investigate the molecular mechanisms of human sarcopenia and skeletal muscle regeneration, we analysed the transcriptional profiles of the skeletal muscle biopsies from healthy older (*N* = 25) and younger (*N* = 26) adult men and women in GSE8479 database, which was obtained from the Gene Expression Omnibus (GEO, https://www.ncbi.nlm.nih.gov/geo/query/acc.cgi). The Volcano plot was performed to show the upregulated and downregulated transcriptional profiles related to sarcopenia. We found that expression of *Creg1* mRNA was dramatically upregulated in older adults compared with young adults; however, after exercise training, *Creg1* mRNA expression was downregulated in older adults, markedly reversed back to that of younger levels (Figure S1A,B). Then, C2C12 myoblast cell was treated with differentiation medium (DM) for 4 days, the expression levels of CREG1 were markedly increased during myoblast differentiation (Figure S1C).

Further, we determined whether CREG1 participated in skeletal muscle regeneration by assessing CREG1 expression during regeneration in a cardiotoxin (CTX)‐induced injury and regeneration model.^17^ Acute muscle injury was induced by injecting CTX into the mice TA muscle (Figure S1D). We analysed the dynamic expression of CREG1 during regeneration following injury. At 3 and 7 days post‐injury (dpi), the expression levels of *Creg1*, *Pax7*, *Myod1*, *MyoG*, and *Myf5* were markedly elevated in quantitative real‐time PCR (RT‐PCR) and western blot (Figure S1E,F) tests; at 14 dpi, their expression levels were gradually restored. These results strongly indicated that CREG1 may be associated with skeletal muscle regeneration.

### CREG1 knockdown impaired skeletal muscle regeneration following injury

**Figure 1:**
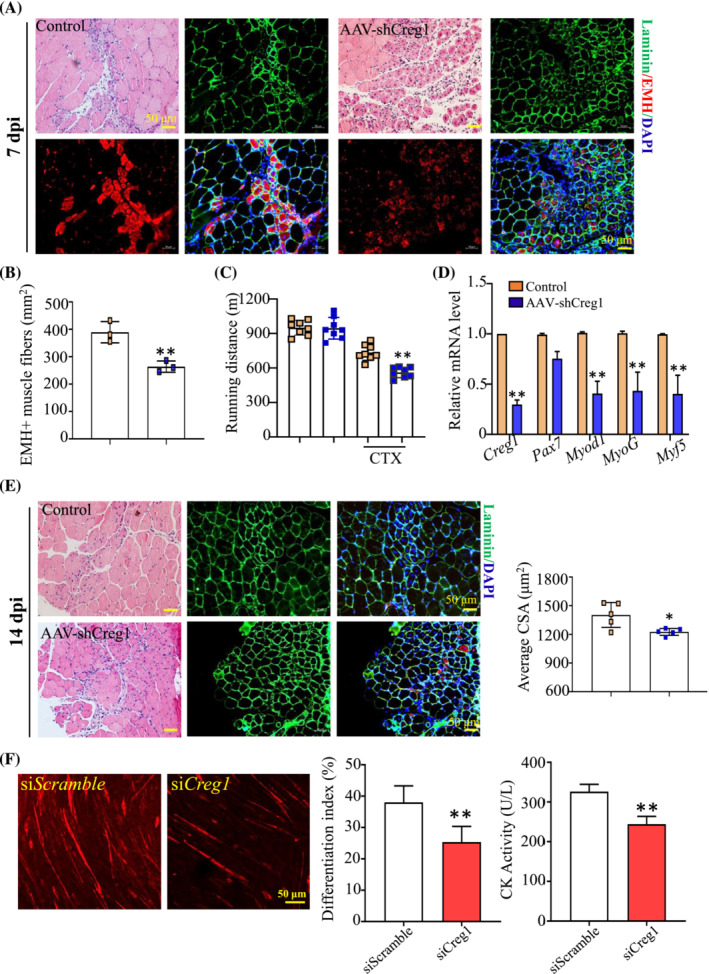
CREG1 knockdown impeded skeletal muscle regeneration after injury. (A, B) Representative H&E staining examined the regeneration of TA muscle at 7 dpi, scale bars: 50 μm. EMH^+^ muscle fibres were detected using IHC staining in TA muscle at 7 dpi, and quantified, scale bars: 50 μm. *N* = 3. (C) Running distance. *N* = 8. (D) RT‐PCR analysis revealed the expression of *Creg1*, *Pax7*, *Myod1*, *MyoG*, and *Myf5* mRNA in TA muscle at 7 dpi. *N* = 3. (E) Representative H&E staining examined regeneration of TA muscle at 14 dpi, scale bars: 50 μm. Laminin was detected by using IHC staining in TA muscle at 14 dpi, scale bars: 50 μm. Quantification cross‐sectional area (CSA). *N* = 5. (F) MyHC IHC staining analysis and quantification revealed the cell differentiation index and creatine kinase (CK) activity, *n* = 5. For all statistical plots, data are shown as mean ± SEM, **P < 0.01, *P < 0.05. Statistical significance was determined by Student's *t* test. CREG1, cellular repressor of E1A‐stimulated genes 1; CSA, cross‐sectional area; CTX, cardiotoxin; dpi, days post‐injury; EMH, embryonic myosin heavy chain (MYH3); H&E, haematoxylin and eosin; TA, tibialis anterior.

To determine whether CREG1 is critical for skeletal muscle regeneration, we generated Creg1 knockdown mice using adeno‐associated virus serotype 9 encoding shCreg1 (AAV‐shCreg1) (Figure S2A). RT‐PCR and western blot analysis revealed efficient reduction of CREG1 transcripts and protein levels in TA and GAS muscles (Figure S2B–D). As shown in Figure S2E, AAV‐shCreg1 mice did not show obvious alteration in histological analysis of TA muscles or differences in cross‐sectional area (CSA). We then explored the regenerative potential of CREG1 knockdown skeletal muscles in response to CTX injection, a well‐known established model of acute skeletal muscle injury, at 3, 7, and 14 dpi. At 3 dpi, haematoxylin and eosin staining showed that many muscle fibres were necrotic in TA muscles of both AAV‐shCreg1 mice and Control mice (Figure S2F). However, Control mice regenerating muscle contained many more newly formed fibres in immunofluorescent staining (IHC) against EMH (myosin heavy chain, MYH3), which is a marker of regenerated fibres. This difference was also observed at 7 dpi. While well‐restored muscle structure with new fibres was observed in Control mice, a much smaller number of regenerated fibres and poorly restored structure was seen in AAV‐shCreg1 mice (Figure 1A,B), and the running distance of AAV‐shCreg1 mice was shorter than those of Control mice (Figure 1C). At 14 dpi, while more immature muscle fibres remained in AAV‐shCreg1 mice, muscle structure and CSA was restored in Control mice to close to normal size (Figure 1E). Consistently, at 7 dpi, the expression of genes important for myogenesis, including *Myod1*, *MyoG*, and *Myf5*, was lower in the regenerating muscle of AAV‐shCreg1 compared with Control mice, whereas *Pax7* was not different between them (Figure 1D). These results indicated that CREG1 knockdown attenuated muscle regeneration, especially myogenic differentiation.

Muscle satellite cells are known as the primary contributor of skeletal muscle regeneration. We evaluated the potential role of CREG1 in satellite cells during myogenesis, CREG1 expression was reduced in C2C12 cells infected with *Creg1* siRNA (named si*Creg1*) compared with the control (named si*Scramble*) (Figure S3A,B). We revealed the differentiation capacities of siCreg1 C2C12 cells in differentiation medium (DM) for 4 days using MyHC staining (Figure 1F). The results showed that the reduction of CREG1 resulted in the decrease of cell differentiation index and Creatine Kinase (CK) activity. These findings indicated that loss of CREG1 impaired the differentiation capacity of satellite cells.

### Muscle satellite cells CREG1 over‐expression enhanced myogenic regeneration following injury

**Figure 2:**
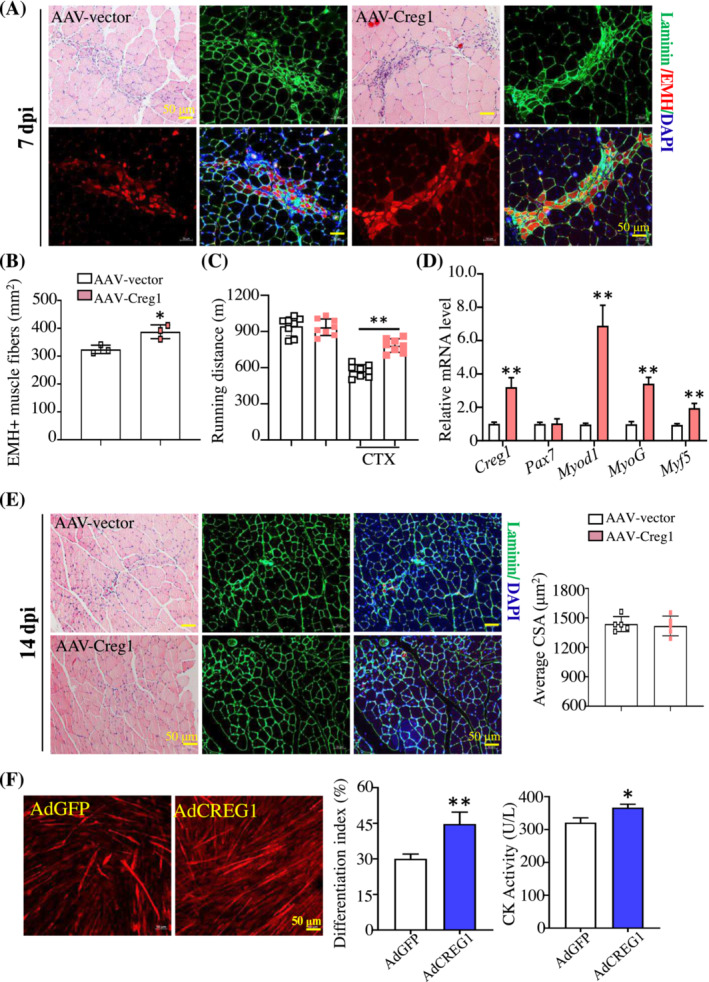
Over‐expression of CREG1 of muscle satellite cells improved skeletal muscle regeneration following injury. (A, B) Representative H&E staining examined regeneration of TA muscle from AAV‐vector and AAV‐Creg1 mice at 7 dpi, scale bars: 50 μm. EMH^+^ muscle fibres were detected using IHC staining in TA muscle at 7 dpi, and quantified, scale bars: 50 μm. *n* = 3. (C) Running distance. *n* = 8. (D) RT‐PCR analysis revealed the expression of *Creg1*, *Pax7*, *Myod1*, *MyoG*, and *Myf5* mRNA in TA muscle at 7 dpi. *n* = 3. (E) Representative H&E staining examined regeneration of TA muscle from AAV‐vector and AAV‐Creg1 mice at 14 dpi, scale bars: 50 μm. Laminin was detected by using IHC staining in TA muscle at 14 dpi, scale bars: 50 μm. Quantification cross‐sectional area (CSA). *n* = 5. (F) MyHC IHC staining analysis and quantification revealed the cell differentiation index and creatine kinase (CK) activity between AdGFP and AdCREG1 C2C12 cells, *n* = 5. For all statistical plots, data are shown as mean ± SEM, **P < 0.01, *P < 0.05. Statistical significance was determined by Student's *t* test. CREG1, cellular repressor of E1A‐stimulated genes 1; CSA, cross‐sectional area; CTX, cardiotoxin; dpi, days post‐injury; EMH, embryonic myosin heavy chain (MYH3); H&E, haematoxylin and eosin; TA, tibialis anterior.

To elucidate whether overexpression of CREG1 could improve myogenic regeneration, muscle satellite cell specific Creg1 overexpression mice (AAV‐Creg1) were generated (Figure S4A). Both immunofluorescent staining and RT‐PCR (Figure S4B,C) analysis showed that the expression of CREG1 was significantly upregulated in the satellite cells of TA and GAS muscles. As shown in Figure S4D,E, compared with AAV‐vector, AAV‐Creg1 mice did not show significantly alteration in histological analysis of TA muscles or differences in CSA. At 7 dpi, better restoration of muscle structure with less necrotic muscle fibres and more regular shaped muscle fibres was observed in AAV‐Creg1 mice (Figure 2A,B), and the running distance of AAV‐Creg1 mice was longer than those of AAV‐vector mice (Figure 2C). Moreover, in TA muscles of AAV‐Creg1 mice, the mRNA level of *Myod1*, *MyoG* and *Myf5* was obviously increased (Figure 2D). At 14 dpi, we found that there were no obvious differences in muscle structure and CSA between AAV‐Creg1 and AAV‐vector mice (Figure 2E). In vitro, we evaluated the differentiation capacities of AdCREG1 C2C12 cells in DM for 4 days using MyHC staining (Figure S3C). The results showed that CREG1 overexpression could upregulate cell differentiation index (Figure 2F). To further evaluate the function of differentiated muscle tubes, the CK activity of differentiated muscle tubes was detected. The results showed that CK activity was increased in AdCREG1 group compared with that in the AdGFP group. These findings suggested that CREG1 over‐expression in muscle satellite cells significantly promoted the muscle regeneration after injury.

### CREG1 negatively regulated C‐CBL expression through ITCH in vitro and in vivo

**Figure 3:**
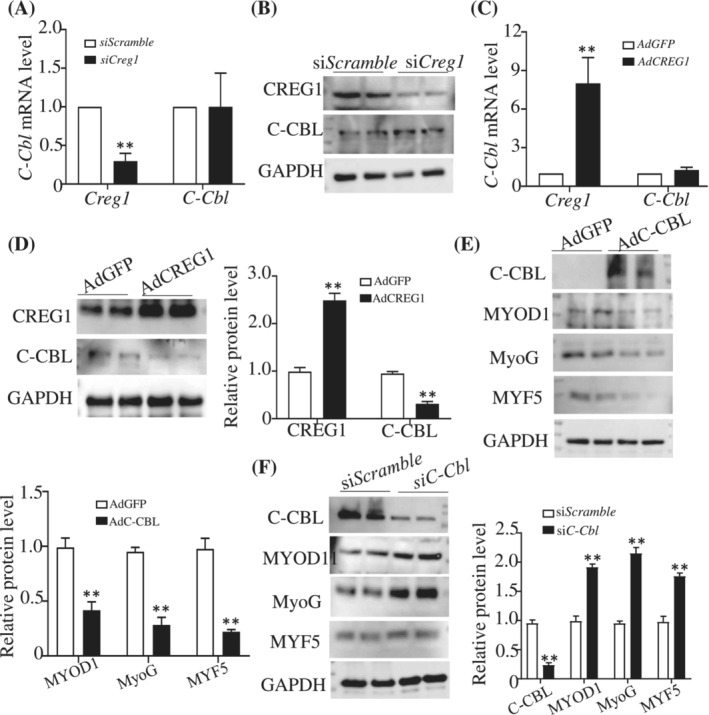
CREG1 negatively regulated C‐CBL expression in C2C12 cells. (A) Real‐time PCR showed the expression of *Creg1* and *C‐Cbl* in the si*Scramble* and si*Cregl* group. *n* = 3. (B) Western blot analysis revealed expression of CREG1 and C‐CBL protein, and quantification. *n* = 3. (C, D) Real‐time PCR and Western blot showed the CREG1 and C‐CBL levels in AdGFP and AdCREG1 group. *n* = 3. (E) Western blot analysis showed the expression of C‐CBL, MYOD1, MyoG, and MYF5 protein in AdGFP and AdC‐CBL group, *n* = 3. (F) Western blot analysis showed the expression of C‐CBL, MYOD1, MyoG, and MYF5 protein in si*Scramble* and si*C‐Cbl* group. *n* = 3. Data are shown as mean ± SEM, **P < 0.01. Statistical significance was determined by Student's *t* test.

To clarify how CREG1 modulated skeletal muscle regeneration, we screened out the C‐CBL protein using Mud‐PIT mass spectrometric analysis (Figure S5A–C). C‐CBL is an E3 ubiquitin protein ligase localized to the cytoplasm, which can inhibit the differentiation of osteoblasts, neuroblasts, oligodendrocytes, and other cells,^18^, ^19^, ^20^, ^21^ but it has not been reported in the skeletal muscle. As shown in Figure 3A,B, the results showed that expression of C‐CBL protein was increased in si*Creg1* C2C12 cells compared with the si*Scramble* group, and decreased in AdCREG1 C2C12 cells compared with the AdGFP group (Figure 3C,D). Furthermore, to detect the effect of C‐CBL on skeletal muscle differentiation, western blot analysis revealed that skeletal muscle differentiation markers, including MYOD1, MyoG and MYF5, were downregulated in C‐CBL‐overexpressing cells (AdC‐CBL) (Figure 3E); however, they were upregulated in C‐CBL‐deficient cells (si*C‐Cbl*) (Figure 3F). These results indicated that CREG may improve skeletal muscle differentiation by negatively regulating C‐CBL.

**Figure 4:**
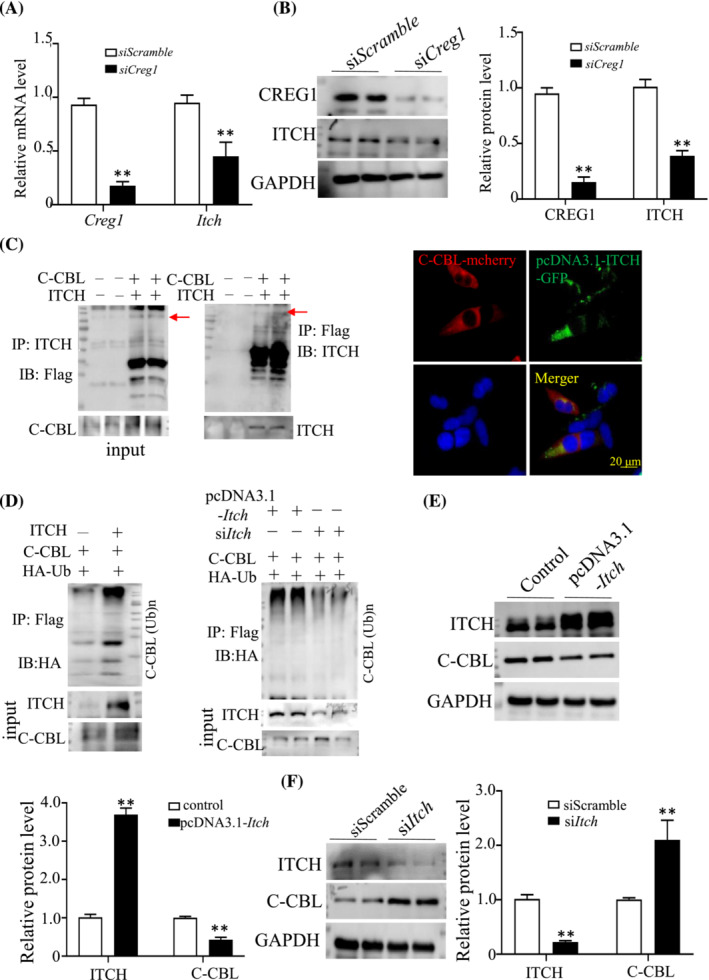
CREG1 regulated C‐CBL through ITCH. (A, B) Real‐time PCR and western blot showed the CREG1 and ITCH levels in the si*Scramble* and si*Cregl* C2C12 cells group. *n* = 3. (C) Co‐immunoprecipitation (co‐IP) analysis to detect interaction between C‐CBL and ITCH in C2C12 cells, IHC staining showed the colocalization between ITCH and C‐CBL when transfected with C‐Cbl mcherry and pcDNA3.1‐*Itch*‐GFP plasmids in C2C12 cells. *n* = 5. (D) Co‐IP analysis to detect ubiquitination expression. *n* = 3. (E) Western blot analysis showed the ITCH and C‐CBL protein expression in ITCH over‐expression C2C12 cells. (F) Western blot analysis showed the ITCH and C‐CBL protein expression in ITCH silenced C2C12 cells, *n* = 3. Data are shown as mean ± SEM, **P < 0.01. Statistical significance was determined by Student's *t* test. CREG1, cellular repressor of E1A‐stimulated genes 1.

C‐CBL mRNA expression was not changed by CREG1. To elucidate how CREG1 negatively modulated C‐CBL protein, we predicted some E3 ubiquitin ligases that may interact with C‐CBL in a comprehensive resource platform UbiBrowser 2.0 (http://ubibrowser.bio‐it.cn/ubibrowser_v3/), especially NEDD4, FBXW7, SMURF1, SMURF2, WWP1, WWP2, and ITCH (Figure S6A). We found that ITCH mRNA and protein levels were decreased in si*Creg1* C2C12 cells compared with si*Scramble* group (Figure 4A,B). Co‐IP between ITCH and C‐CBL showed that ITCH interacted with C‐CBL and degraded it, the colocalization between ITCH and C‐CBL was observed by IHC when transfected with *C‐Cbl mcherry* and pcDNA3.1‐*Itch*‐GFP plasmids in C2C12 cells (Figure 4C). ITCH overexpression enhanced ubiquitination of C‐CBL; however, ITCH silencing attenuated ubiquitination of C‐CBL in C2C12 myotubes (Figure 4D). Overexpression of ITCH in C2C12 cells was found to degrade C‐CBL protein, but did not affect C‐CBL mRNA levels (Figure 4E, Figure S6B). Conversely, silencing of ITCH increased C‐CBL protein (Figure 4F). These results suggested that CREG1 negatively regulated C‐CBL through ITCH.

### C‐CBL was an E3 ubiquitin ligase targeting AMPKa1 for ubiquitin‐dependent degradation

**Figure 5:**
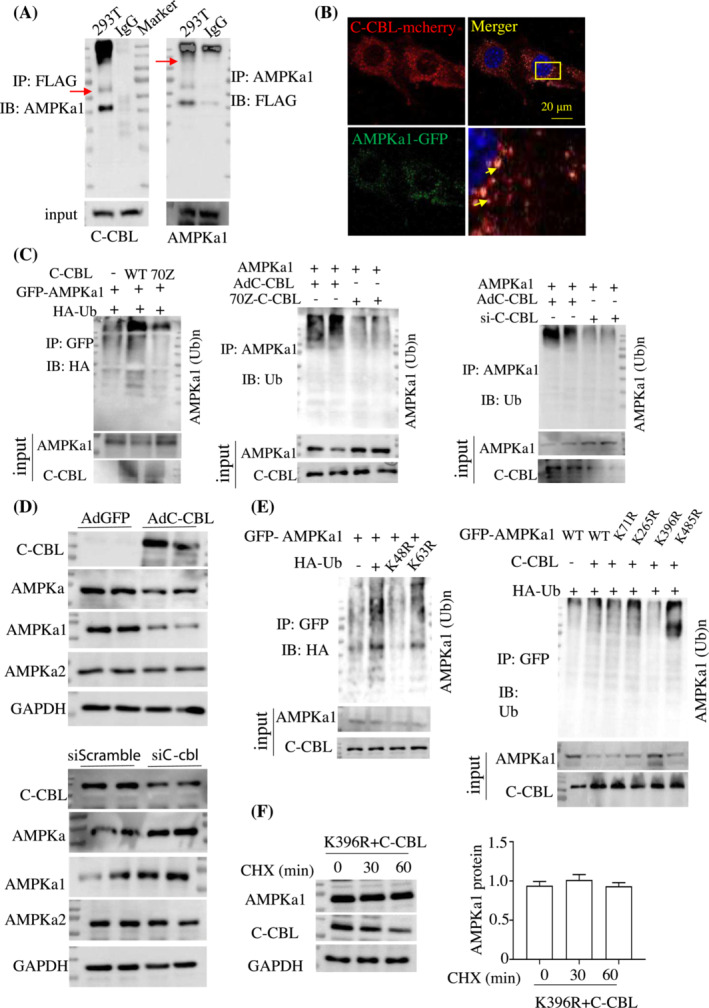
C‐CBL was targeting AMPKa1 for ubiquitin‐dependent degradation. (A) The interaction between C‐CBL and AMPKa1 in HEK293T cells was evaluated by co‐immunoprecipitation (co‐IP). *n* = 3. (B) Representative images of immunofluorescent staining of C‐CBL and AMPKa1 showing their co‐localization in C2C12 cells, *n* = 5, scale bars: 20 μm. (C) Co‐IP analysis in wildtype (WT), the 70Z mutant of C‐CBL and knockdown of C‐CBL in HEK293T cells. (D) Western blot analysis showed the C‐CBL, AMPKa, AMPKa1, and AMPKa2 expression in AdGFP, AdC‐CBL, si*Scramble*, and si*C‐Cbl* group. (E) K71R, K265R, or K485R mutants of AMPKa1 were evaluated in HEK293T cells. (F) Representative western blots showing the protein expression of K396R mutants of AMPKa1 in HEK293T cells at the indicated time points after cycloheximide (CHX, 20 μg/mL) treatment. *n* = 3. Data are shown as mean ± SEM, **P < 0.01. Statistical significance was determined by Student's *t* test. ns, no significance between the two indicated groups by one‐way ANOVA. ANOVA, analysis of variance; CREG1, cellular repressor of E1A‐stimulated genes 1.

Interestingly, C‐CBL‐interacting proteins were isolated from C2C12 cells by expressing flag‐tagged C‐CBL. We identified AMPKa1 that interacted with C‐CBL in immunopurified CREG1 complexes using Mud‐PIT mass spectrometric analysis (Figure S7A). AMPKa1, the dominant AMPKα isoform in muscle satellite cells, facilitates muscle regeneration.^22^, ^23^ Western blot analysis showed that the expression of MYOD1, MyoG, and MYF5 protein was decreased when Ampka1 was silenced in C2C12 myotubes (Figure S7B). As shown in Figure 5A, we confirmed that C‐CBL interacted with AMPKa1 using the Co‐IP assay in the HEK293T cells. In C2C12 myotubes, the colocalization between C‐CBL and AMPKa1 was profoundly revealed by IHC when transfected with *C‐Cbl‐*mcherry and *Ampka1*‐GFP plasmids (Figure 5B). Furthermore, the Co‐IP assay revealed that C‐CBL interacted with the C‐terminus of AMPKa1 (392–598 aa) (Figure S8A‐B). These results implied that AMPKa1 was a direct substrate of C‐CBL.

Further, we found that overexpression of C‐CBL, but not its E3 ligase inactive mutant (C‐CBL 70Z),^24^, ^25^ boosted the ubiquitination of AMPKa1; however, knockdown of C‐CBL in C2C12 myotubes attenuated AMPKa1 ubiquitination (Figure 5C). These results indicated that C‐CBL functioned as an E3 ligase to catalyse ubiquitination of the AMPKa1 protein. We then examined whether C‐CBL regulated AMPKa1 protein stability. As shown in Figure 5D, when C‐CBL was overexpressed in C2C12 cells, AMPKa1 protein expression significantly decreased, but no significant change was observed in mRNA level (Figure S8C), AMPKa2 protein expression was also not changed, when C‐CBL was silenced in C2C12 cells, AMPKa1 protein expression remarkably increased; however, AMPKa2 protein expression was not changed. In addition, the downregulation of AMPKa1 by C‐CBL was blocked by the proteasome inhibitor MG132 (10 μmol/L) for 24 h (Figure S8D). We then constructed two mutants of ubiquitin, lysine 48 or lysine 63 to arginine (K48R or K63R),^26^ and found that C‐CBL‐induced AMPKa1 ubiquitination occurred through K48‐linked chains rather than K63‐linked chains (Figure 5E), suggesting that C‐CBL‐mediated ubiquitination targets AMPKa1 for degradation. Four potential mutation ubiquitination sites on AMPKa1 (Figure S8E)^27^ revealed that lysine 396 to arginine (K396R), but not lysine 71 (K71R), lysine 265 (K265), or lysine 485 (K485), markedly attenuated ubiquitination of AMPKa1 by C‐CBL (Figure 5E). Only AMPKa1 K396R was resistant to C‐CBL‐mediated degradation (Figures 5F and Figure S8F). These findings indicated that C‐CBL‐mediated AMPKa1 ubiquitination was attributed to the K48‐linked polyubiquitination of AMPKa1 at K396 and that this modification played an important role in the regulation of AMPKa1 protein stability.

### Skeletal mature myofibre‐specific CREG1 deletion impaired muscle regeneration

**Figure 6:**
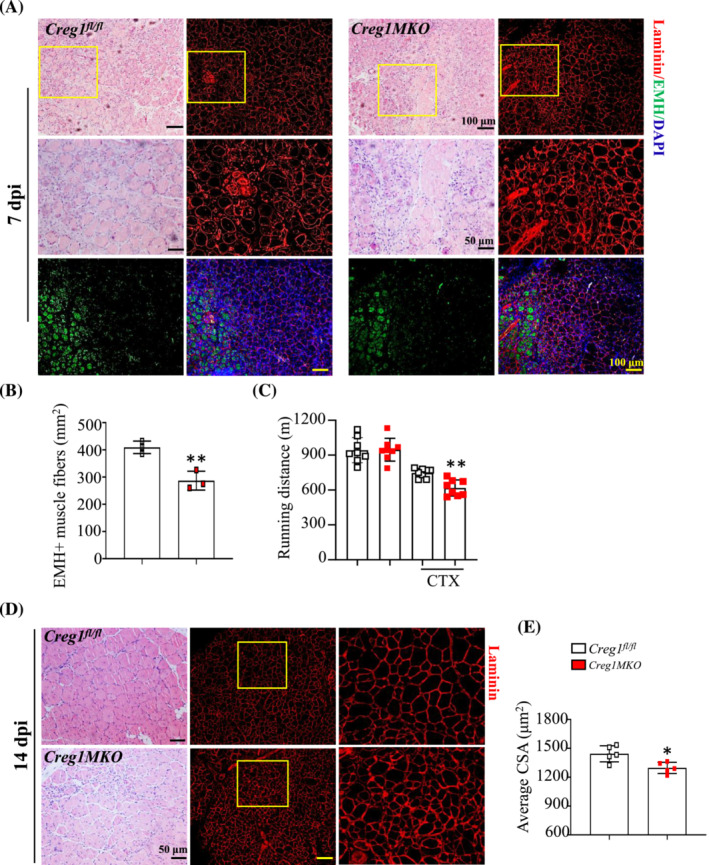
CREG1 deletion of mature myofiber impaired skeletal muscle regeneration after injury. (A, B) Representative H&E staining examined the regeneration of TA muscle at 7 dpi, scale bars: 50 and 100 μm. EMH^+^ muscle fibres were detected by using IHC staining in TA muscle at 7 dpi, and quantified, scale bars: 100 μm. *n* = 3. (C) Running distance. *n* = 8. (D, E) Representative H&E staining examined regeneration of TA muscle; laminin was detected by using IHC staining in TA muscle at 14 dpi. Quantification cross‐sectional area (CSA). *n* = 5. Scale bars: 50 and 100 μm. For all statistical plots, data are shown as mean ± SEM, **P < 0.01, *P < 0.05. Statistical significance was determined by Student's *t* test. CREG1, cellular repressor of E1A‐stimulated genes 1; CSA, cross‐sectional area; CTX, cardiotoxin; dpi, days post‐injury; EMH, embryonic myosin heavy chain (MYH3); H&E, haematoxylin and eosin; TA, tibialis anterior.

Mature myofibres also played an important role in regulating muscle stem cells microenvironment and regenerative capacity.^12^ To further investigate the effect of CREG1 deletion in mature myofibres on skeletal muscle regeneration, we generated a myofibre‐specific *Creg1* knockout mouse (*Creg1MKO*) and control mice *Creg1* ^*flox/flox*^ (*Creg1* ^*fl/fl*^) using CRISPR/Cas9‐mediated genome editing, and genotyping was identified (Figure S9A,B). RT‐PCR and western blot revealed CREG1 transcripts and protein levels were markedly downregulated in TA muscles (Figure S9C,D). We found that 8‐week‐old *Creg1MKO* mice were indistinguishable from *Creg1* ^*fl/fl*^ mice, based on appearance, weight, and histological analysis of TA muscles (Figure S9E,F). Next, as shown in Figure 6A–C, we found that more newly formed muscle fibres, better muscle structure restoration and longer running distance were observed in *Creg1* ^*fl/fl*^ than *Creg1MKO* mice at 7 dpi. Consistently, there was some immature muscle fibres remained in the TA muscle of *Creg1MKO* mice, and CSA was markedly decreased compared with those from *Creg1* ^*fl/fl*^ mice muscle at 14 dpi (Figure 6D,E). These results suggested that CREG1 deficiency of mature myofibre impaired muscle regeneration.

**Figure 7:**
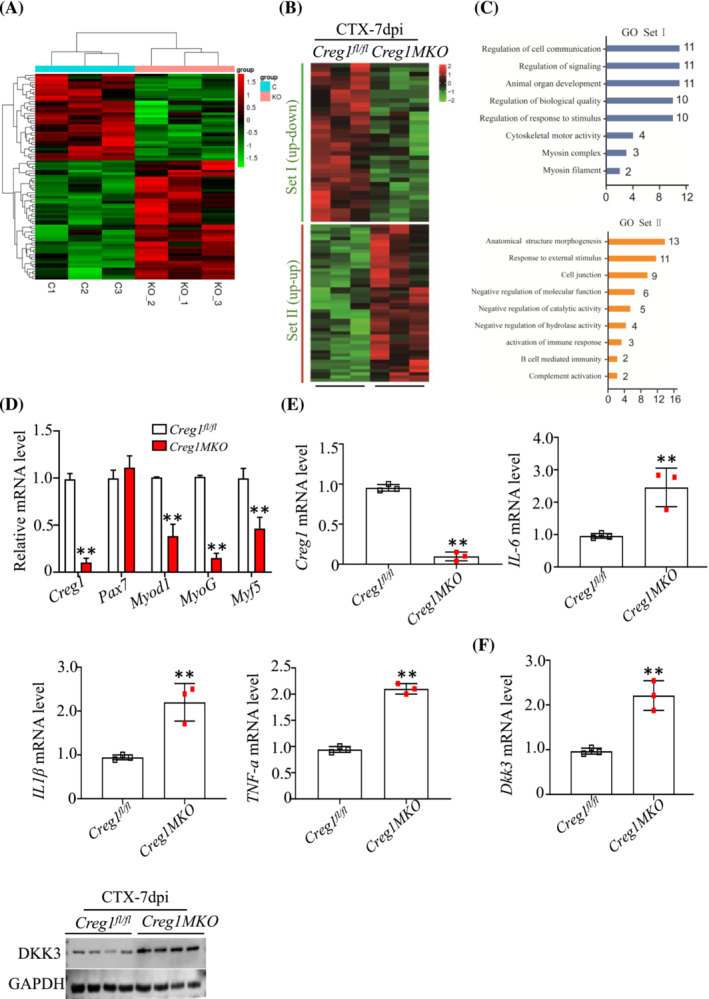
Deficiency of CREG1 in mature myofiber prevented muscle regeneration through excessive inflammation and Dkk3 upregulation. (A–C) Heatmap representation and GO analysis of genes in set I (CTX up and Creg1MKO down) and set II (CTX up and *Creg1MKO* up). *n* = 3 mice per group. (D) RT‐PCR analysis revealed the expression of *Creg1*, *Pax7*, *Myod1*, *MyoG*, and *Myf5* mRNA in TA muscle at 7 dpi. *n* = 3. (E) RT‐PCR analysis revealed the expression of *Creg1*, *IL6*, *IL1β*, and *TNFa* mRNA in TA muscle at 7 dpi. *n* = 3. (F) Real‐time PCR and western blot showed the DKK3 mRNA and protein levels. *n* = 3. For all statistical plots, data are shown as mean ± SEM, **P < 0.01, *P < 0.05. Statistical significance was determined by Student's *t* test. CREG1, cellular repressor of E1A‐stimulated genes 1; CSA, cross‐sectional area; dpi, days post‐injury; EMH, embryonic myosin heavy chain (MYH3); H&E, haematoxylin and eosin; TA, tibialis anterior.

We performed RNA sequencing analysis to examine the gene expression profiles in *Creg1* ^*fl/fl*^ and *Creg1MKO* mice TA muscles following CTX injection for 7 dpi. These genes were divided into two groups: Set I (CTX up and *Creg1MKO* down) and Set II (CTX up and *Creg1MKO* up). As shown in Figure 7A–C, Gene Ontology (GO) analysis showed that the genes were enriched in muscle development and function in Set I section, while genes in Set II section were enriched in molecular dysfunction and inflammation‐related pathways. In damaged muscle, transcription levels of *Myod1*, *MyoG*, and *Myf5* were lower in *Creg1MKO* than in *Creg1* ^*fl/fl*^ mice TA muscle (Figure 7D). Previous studies have reported that mature myofibre inactivation often impairs muscle regeneration by disturbance muscle satellite cells environment.^12^ As for inflammation, transcription levels of proinflammatory factors *IL6*, *IL‐1β* and *TNF‐a* were markedly upregulated in *Creg1MKO* than in *Creg1* ^*fl/fl*^ mice TA muscle (Figure 7E). We also found that *Dickkopf 3* (*Dkk3*) gene was markedly upregulated in *Creg1MKO* TA muscle, which plays an important role in negative regulating muscle regeneration. RT‐PCR and western blot analysis confirmed that the expression level of DKK3 mRNA and protein was significantly elevated (Figure 7F). These findings suggested that deficiency of CREG1 in mature myofibre declined muscle regeneration capacity following injury.

### C‐Cbl knockdown could improve Creg1MKO mice muscle regeneration in vivo

**Figure 8:**
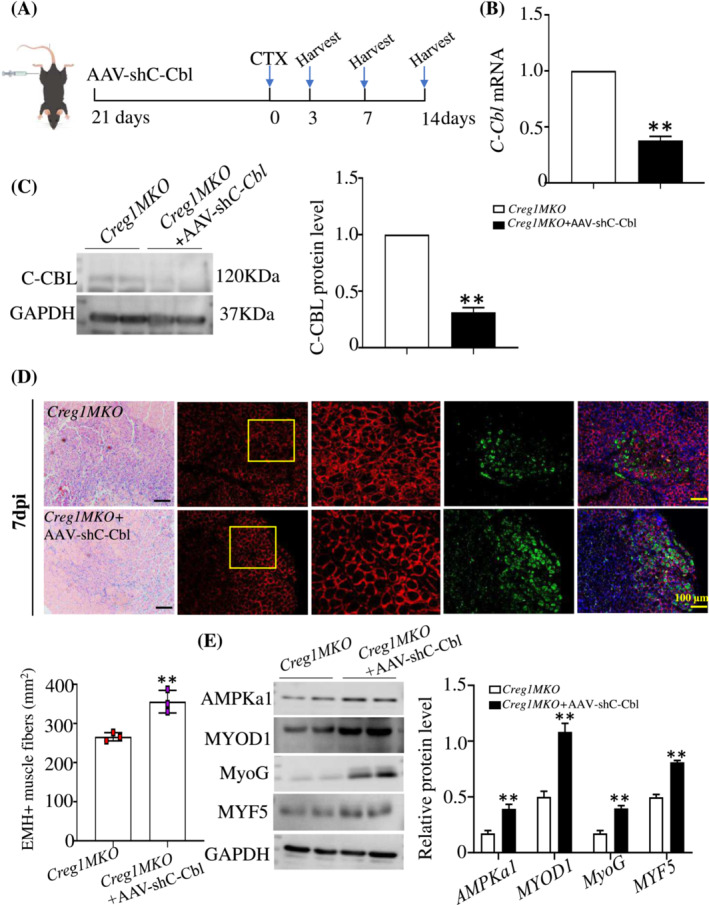
AAV*‐*shC‐Cbl improved *Creg1MKO* mice skeletal muscle regeneration. (A) Schematic of CTX injury model following AAV‐shC‐Cbl injection through muscle location. (B, C) RT‐PCR and western blot analysis detected the expression of C‐CBL in TA muscle, *n* = 3. (D) Representative H&E staining examined regeneration of TA muscle from *Creg1MKO* mice at 7 dpi, scale bars: 100 μm. EMH^+^ muscle fibres detected by IHC staining in TA muscle and quantification, *n* = 3. (E) Expression of AMPKa1, MYOD1, MyoG, and MYF5 protein was detected by using western blot and quantification analysis. *n* = 3. For all statistical plots, data are shown as mean ± SEM, **P < 0.01. Statistical significance was determined by Student's *t* test. CREG1, cellular repressor of E1A‐stimulated genes 1; CTX, cardiotoxin; dpi, days post‐injury; EMH, embryonic myosin heavy chain (MYH3); H&E, haematoxylin and eosin; TA, tibialis anterior.

Additionally, we assessed whether C‐CBL knockdown could improve the detrimental effects of CREG1 deficiency on skeletal muscle regeneration in vivo. The TA muscles of *Creg1MKO* mice were injected with control or AAV‐shC‐Cbl (Figure 8A). C‐CBL expression in TA muscles was silenced by AAV‐shC‐Cbl injection through muscle location, and determined by RT‐PCR and western blot analysis (Figure 8B,C). Silencing C‐CBL in the TA muscles of *Creg1MKO* mice significantly improved skeletal muscle regeneration induced by CTX intramuscular injury. As shown in Figure S10, at 3 dpi, *Creg1MKO* mice injected with AAV‐shC‐Cbl regenerating muscle contained much more EMH^+^ muscle fibres by IHC staining. At 7 dpi, better restoration of muscle structure with less necrotic muscle fibres and more regular shaped muscle fibres was observed in *Creg1MKO* mice injected with AAV‐shC‐Cbl (Figure 8D), and myogenic differentiation gene expression was upregulated (Figure 8E). These results indicated that C‐CBL knockdown rescued muscle regeneration capacity following injury in CREG1 knockout mature myofibres.

## Discussion

In the present study, we identified that CREG1 was a muscle protective factor in skeletal muscle regeneration for several reasons: (1) CREG1 deficiency of muscle satellite cells significantly impeded myogenic differentiation and muscle regeneration; overexpression of CREG1 in muscle satellite cells could accelerate the skeletal muscle regeneration process; (2) the protective role of CREG1 in skeletal muscle regeneration was dependent on negatively regulating C‐CBL; further mechanistic investigations revealed that C‐CBL inhibited satellite cells differentiation by directly degrading the substrate protein AMPKa1, affecting the AMPKa1 signalling pathway; (3) CREG1 ablation in muscle mature myofibre inhibited muscle satellite cells differentiation and muscle regeneration following injury, which was probably through disturbing muscle satellite cells microenvironment (Figure S10C). Taken together, our findings provide promising evidence of how CREG1 acts as a positive regulator of skeletal muscle regeneration.

Previous studies have identified that CREG1 is a secreted glycoprotein that is conserved throughout evolution and is expressed in many tissues, including the spleen, liver, kidney, lung, heart, fat tissue, and skeletal muscle. The expression level of CREG1 is low in undifferentiated embryonic stem cells and embryonal cells and is rapidly upregulated upon cellular differentiation.^13^ These results suggest that CREG1 plays a vital role in development and normal physiology. Studies have revealed that CREG1 could induce differentiation as a soluble factor in embryonal carcinoma cells; overexpression of CREG1 augmented retinoic acid‐induced differentiation of embryonal carcinoma cells into the neuronal lineage; CREG1 expression was induced during the differentiation of human vascular smooth muscle cells; using gain‐ and loss‐of‐function analysis, we have also shown that CREG1 induced cardiomyogenic differentiation from embryonic stem cells.^14^, ^28^ These research results verified that CREG1 played an important role in promoting cell differentiation; however, its precise role in myogenic differentiation, especially in regeneration, is not clear. Here, our data revealed that CREG1 knockdown by AAV impeded myogenic differentiation and skeletal muscle regeneration after injury in vivo. Further, muscle satellite cells CREG1 over‐expression accelerated CTX‐induced skeletal muscle regeneration process compared with control mice. Thus, these findings demonstrated that CREG1 positively regulated skeletal muscle regeneration.

To elucidate the molecular mechanism of CREG1 regulating muscle satellite cells differentiation and skeletal muscle regeneration, we used the Mud‐PIT mass spectrometric analysis approach to identify C‐CBL as a potential interactant for CREG1 in C2C12 cells. C‐CBL was identified as a gene encoding a RING finger E3 ubiquitin ligase, regulating the differentiation of osteoblasts, neuroblasts, oligodendrocytes, and myeloblastic leukaemic cells.^29^, ^30^ Loss‐of‐function and gain‐of‐function experiments in vitro provided evidence that C‐CBL also played a negative role in myogenic differentiation. Furthermore, the lack of CREG1 resulted in increased expression of the C‐CBL protein at the post‐transcriptional level, but not at the transcriptional level; however, we found that CREG1 may not directly interact with C‐CBL. Therefore, we used the UbiBrowser 2.0 database platform (http://ubibrowser.bio‐it.cn/ubibrowser_v3/) to predict E3 ubiquitin ligase activity when C‐CBL was the substrate protein. The predicted results showed that Nedd4, Sumurf2, and ITCH may participate in the degradation of C‐CBL as E3 ubiquitin enzymes. Our detection revealed that ITCH overexpression could significantly downregulate the protein level of C‐CBL, and ITCH silenced could significantly increase the protein level of C‐CBL. Studies have found that the ITCH protein could interact with CBL in several cell types and regulate CBL ubiquitylation degradation^31^; however, whether the combination of the two proteins exists in satellite cells has not yet been investigated. We verified the interaction via Co‐IP and colocalization of ITCH1 and C‐CBL in C2C12 cells by confocal imaging. ITCH overexpression increased the ubiquitination level and led to increased degradation of substrate CBL protein. We also demonstrated that CREG1 regulates the expression of ITCH at the transcriptional and translational levels in vivo and in vitro. These data suggest that CREG1 modulates C‐CBL expression by regulating ITCH.

C‐CBL, as an E3 ubiquitin ligase, recognizes activated target proteins and ubiquitinates them.^32^, ^33^ We used the Mud‐PIT mass spectrometric analysis approach to reveal that C‐CBL may interact with AMPKa1 in C2C12 cells. Recent studies have shown that AMPKα1, the dominant AMPKα isoform in satellite cells, facilitates myogenic differentiation and skeletal muscle regeneration following injury. AICAR, an AMPK activity agonist, successfully improved muscle regeneration in obese mice^2^, ^34^; however, AMPKα1 knockout in satellite cells abolished the positive effect of AICAR administration on skeletal muscle regeneration. These results confirm that AMPKα1 plays a critical role in skeletal muscle regeneration following injury. To further investigate how C‐CBL modulates AMPKa1 protein stability, in our study, AMPKα1 was demonstrated to interact with E3 ubiquitin ligase C‐CBL, and C‐CBL can stabilize AMPKα1 by catalysing K48‐linked polyubiquitination. K48‐linked chains, the most abundant linkage type, are involved in the delivery of substrate proteins to the proteasome for degradation. Moreover, the C‐CBL mutant (70Z) or silencing in vitro and in vivo improved satellite cell differentiation and muscle regeneration by destabilizing AMPKα1 via inhibition of K48‐linked ubiquitination. Furthermore, among the four mutants, only AMPKa1 K396R prevented C‐CBL‐mediated degradation. Another study reported that oncogenic MAGEA‐TRIM28 ubiquitin ligase targeted AMPKα1 for ubiquitination and proteasome‐mediated degradation in cancer.^35^ In our study, we did not detect MAGEA‐TRIM28 ubiquitin ligase expression with or without CREG1 deficiency following muscle injury. These findings indicate that C‐CBL‐mediated K48‐linked AMPKa1 polyubiquitination plays a novel role in skeletal muscle regeneration in the absence of CREG1.

Skeletal muscle repair is a complex process including inflammatory response, muscle regeneration and remodelling.^36^, ^37^ Muscle satellite cells are defined as the major contributor of muscle regeneration, professor Meng team^12^ identified that mature myofibre played an important role in regulating satellite cells microenvironment and muscle regeneration through the paracrine manner. In the resent study, myofibre‐specific *Creg1* knockout mouse was generated and did not demonstrate alterations in terms of myofibre number and size in 8‐week‐old mice; however, CREG1 deletion decreased the number of type I fibre in skeletal muscles of 9‐month‐old *Creg1MKO* mice. RT‐PCR results showed that expression of MHCI mRNA level was significantly downregulated in *Creg1MKO* mice compared with *Creg1* ^*fl/fl*^ mice at 9‐month‐old.^16^ These findings indicated that the loss of CREG1 may affect the molecules involved in fibre type switching and even cause skeletal muscle atrophy with aging, but we would investigate this further. Following CTX injection, CREG1 deletion in mature myofibres markedly inhibited muscle satellite cells differentiation and muscle regeneration. RNA sequencing analysis suggested that CTX injection in *Creg1MKO* mice led to downregulation of genes in muscle development and upregulation of secreted‐protein encoding genes, especially *Dkk3*. It was demonstrated that DKK3 was abundantly produced and secreted in skeletal muscle^38^, ^39^ and negatively regulates skeletal muscle regeneration through paracrine signalling. As expected, RT‐PCR and western blot data confirmed that the expression of DKK3 mRNA and protein levels were elevated significantly. We speculated that CREG1 deficiency in mature myofibre prevented muscle regeneration through probably promotion of DKK3 production and secretion. Immune cells could communicate with muscle satellite cells and impact the muscle regeneration process. Some studies have demonstrated that perturbation of immune homeostasis impaired muscle regeneration, and persistent inflammation was one reason of muscle repair deficits.^36^ Our data showed that CTX injection at 7 dpi in *Creg1MKO* mice may cause excessive inflammatory response and damage the differentiation capacity of muscle satellite cells. However, the detailed molecular mechanism needs us to explore further.

Taken together, our study uncovers a novel role of CREG1 in muscle satellite cells in modulating muscle regeneration through regulating C‐CBL‐AMPKa1 signalling, and in mature myofibres modulating muscle regeneration through impacting muscle satellite cells microenviroment. These findings suggest that CREG1 may be a potential target for therapeutic intervention in skeletal muscle regeneration following injury.

## Conflict of interests

The authors declare no competing interests.

## Supporting information


**Figure S1.** CREG1 expression was associated with skeletal muscle regeneration. (A‐B) Volcano plot; Heatmap; Quantitation of upregulated and downregulated transcriptional profiles of the skeletal muscle from healthy older (*N* = 25) and younger (*N* = 26) adult men and women in GSE8479 database. (a: older people before exercise; b: older people after exercise) (C) Real‐time PCR and western blot analysis detected expression of CREG1 in C2C12 cells differentiation from 1 to 4 days. (*n* = 3). (D) Schematic of CTX injury model. (E) Real time PCR analysis of Creg1, Pax7, Myod1, MyoG and Myf5 expression in the TA muscles. (*n* = 3). (F) Western blot analysis revealed proteins expression in the TA muscles (n = 3). For all statistical plots, data are shown as mean ± SEM, **p < 0.01, #*p* < 0.05. dpi: days post‐injury; CREG1: cellular repressor of E1A‐stimulated genes 1; TA: tibialis anterior.
**Figure S2.** Generation of Creg1 knockdown mice by adeno‐associated virus serotype 9 (AAV9). (A) A chart described an organization knockdown strategy. (B) Realtime PCR detected Creg1 mRNA expression in tibialis anterior (TA) and Gastrocnemius (GAS). (*n* = 3). (C‐D) Western blot showed the expression of CREG1 in tibialis anterior (TA) and Gastrocnemius (GAS). (n = 3). (E) Representative H&E and immunofluorescent staining analysis of TA muscles in cross‐sectional area (CSA), (*n* = 5). scale bars: 50 μm. (F) Representative H&E staining examined regeneration of TA muscle at 3 dpi, EMH + muscle fibres detected by IHC staining in TA muscle at 3 dpi, scale bars: 50 μm. *n* = 5. For all statistical plots, data are shown as mean ± SEM, **p < 0.01. Statistical significance was determined by Student‘s t test. Dpi: days post‐injury; CREG1: cellular repressor of E1Astimulated genes 1; TA: tibialis anterior; GAS: Gastrocnemius; CSA: cross‐sectional area.
**Figure S3.** CREG1 silence and overexpression in C2C12 cells. (A‐C) Western blot analysis showed expression of CREG1 in loss‐ and gain‐of function approach. For all statistical plots, data are shown as mean ± SEM, **p < 0.01. Statistical significance was determined by Student's t test.
**Figure S4.** Generation of muscle satellite cells specific Creg1 overexpression mice by adeno‐associated virus serotype 9 (AAV9). (A) A chart described an organization overexpression strategy. (B) Immunofluorescent staining analysis examined the expression of CREG1 and Laminin, scale bars: 20 μm. *n* = 5.(C) Real‐time PCR detected Creg1 mRNA expression in tibialis anterior (TA) and Gastrocnemius (GAS). *n* = 3. (D‐E) Representative H&E and immunofluorescent staining analysis of TA muscles in cross‐sectional area (CSA), scale bars: 50 μm. *n* = 5. For all statistical plots, data are shown as mean ± SEM, **p < 0.01. Statistical significance was determined by Student's t test. CREG1: cellular repressor of E1A‐stimulated genes 1; TA: tibialis anterior; GAS: Gastrocnemius; CSA: cross‐sectional area.
**Figure S5.** Mass Spectrometric analysis.
**Figure S6.** ITCH interacted with C‐CBL in C2C12 cells. (A) Some E3 ubiquitin ligases were predicted in UbiBrowser 2.0 database (http://ubibrowser. bio‐it. cn/ubibrowser_v3/). (B) RT‐PCR analysis of C‐Cbl expression, *n* = 3. For all statistical plots, data are shown as mean ± SEM. Statistical significance was determined by Student's t test.
**Figure S7.** C‐CBL interacted with AMPKa1 in C2C12 cells. (A) Mass Spectrometric analysis. (B) Western blot analysis showed expression of AMPKa1, MYOD1, MyoG and MYF5. *n* = 3. For all statistical plots, data are shown as mean ± SEM, **p < 0.01. Statistical significance was determined by Student's t test.
**Figure S8.** C‐CBL is an E3 Ligase of AMPKa1, related to Figure 6. (A‐B) The interaction between C‐CBL‐Flag and AMPKa1‐GFP in HEK293 T cells was evaluated by co‐immunoprecipitation (Co‐IP). (C) RT‐PCR analysis of C‐Cbl and Ampka1 expression, *n* = 3. (D) Western blot analysis showed expression of AMPKa1 with or without MG132 administrated. (E) Candidate ubiquitin sites in AMPKa1. (F) Representative western blot showing the protein expression of wild type, K71R, K265R or K485R mutants of AMPKa1 in HEK293 T cells at the indicated time points after Cycloheximide (CHX, 20 μg/mL) treatment. *n* = 3, data are shown as mean ± SEM, **p < 0.01. Statistical significance was determined by Student's t tes.
**Figure S9.** Generation of creg1 skeletal muscle‐specific knockout mice. (A) A chart described an organization‐specific knockout strategy. (B) Real‐time PCR detected Creg1 gene and Cre gene expression. (C‐D) Real‐time PCR and western blot showed the expression of CREG1 in tibialis anterior (TA) of Creg1 fl/fl and Creg1MKO. *n* = 3. (E) A TA and gastrocnemius (GAS) muscles representative image and quantification from Creg1 fl/fl and Creg1MKO mice. (F) H&E staining examined TA muscle at uninjury, scale bars: 100 μm. Average values of myofiber cross‐sectional areas (CSA). *n* = 5. For all statistical plots, data are shown as mean ± SEM, *p < 0.05, **p < 0.01. Statistical significance was determined by Student's t test. TA: tibialis anterior; GAS: Gastrocnemius; CSA: crosssectional area.
**Figure S10.** C‐CBL knockdown in TA muscle of Creg1MKO mice improved muscle regeneration. (A‐B) Representative H&E staining examined regeneration of TA muscle at 3 dpi, scale bars: 100 μm. EMH + muscle fibres detected by IHC staining in TA muscle at 3 dpi and quantification, scale bars: 100 μm. *n* = 3. (C) A schematic picture. For all statistical plots, data are shown as mean ± SEM, **p < 0.01, ##*p* < 0.01. TA: tibialis anterior.

## References

[1] Luo W , Lin Z , Jiahui C , Genghua C , Siyu Z , Manqing L , et al. TMEM182 interacts with integrin beta 1 and regulates myoblast differentiation and muscle regeneration. J Cachexia Sarcopenia Muscle 2021;12:1704–1723. 34427057 10.1002/jcsm.12767 PMC8718073

[2] Fu X , Zhu M , Zhang S , Foretz M , Viollet B , du M . Obesity impairs skeletal muscle regeneration through inhibition of AMPK. Diabetes 2016;65:188–200. 26384382 10.2337/db15-0647 PMC4686944

[3] Michele DE . Mechanisms of skeletal muscle repair and regeneration in health and disease. FEBS J 2022;289:6460–6462. 35929418 10.1111/febs.16577

[4] Sciorati C , Rigamonti E , Manfredi AA , Rovere‐Querini P . Cell death, clearance and immunity in the skeletal muscle. Cell Death Differ 2016;23:927–937. 26868912 10.1038/cdd.2015.171 PMC4987728

[5] Zhang M , Han Y , Liu J , Liu L , Zheng L , Chen Y , et al. Rbm24 modulates adult skeletal muscle regeneration via regulation of alternative splicing. Theranostics 2020;10:11159–11177. 33042276 10.7150/thno.44389 PMC7532667

[6] Relaix F , Zammit PS . Satellite cells are essential for skeletal muscle regeneration: the cell on the edge returns centre stage. Development 2012;139:2845–2856. 22833472 10.1242/dev.069088

[7] Núñez‐Álvarez Y , Hurtado E , Muñoz M , García‐Tuñon I , Rech GE , Pluvinet R , et al. Loss of HDAC11 accelerates skeletal muscle regeneration in mice. FEBS J 2021;288:1201–1223. 32602219 10.1111/febs.15468

[8] Li H , Malhotra S , Kumar A . Nuclear factor‐kappa B signaling in skeletal muscle atrophy. J Mol Med (Berl) 2008;86:1113–1126. 18574572 10.1007/s00109-008-0373-8 PMC2597184

[9] García‐Prat L , Martínez‐Vicente M , Perdiguero E , Ortet L , Rodríguez‐Ubreva J , Rebollo E , et al. Autophagy maintains stemness by preventing senescence. Nature 2016;529:37–42. 26738589 10.1038/nature16187

[10] Hernández‐Hernández JM , García‐González EG , Brun CE , Rudnicki MA . The myogenic regulatory factors, determinants of muscle development, cell identity and regeneration. Semin Cell Dev Biol 2017;72:10–18. 29127045 10.1016/j.semcdb.2017.11.010 PMC5723221

[11] Ferri P , Barbieri E , Burattini S , Guescini M , D'Emilio A , Biagiotti L , et al. Expression and subcellular localization of myogenic regulatory factors during the differentiation of skeletal muscle C2C12 myoblasts. J Cell Biochem 2009;108:1302–1317. 19830700 10.1002/jcb.22360

[12] Jingya X , Li X , Chen W , Ziyin Z , Yanping Z , Yahui G , et al. Myofiber Baf60c controls muscle regeneration by modulating Dkk3‐mediated paracrine signaling. J Exp Med 2023;220:e20221123. 37284884 10.1084/jem.20221123 PMC10250555

[13] Veal E , Groisman R , Eisenstein M , Gill G . The secreted glycoprotein CREG enhances differentiation of NTERA‐2 human embryonal carcinoma cells. Oncogene 2000;19:2120–2128. 10815803 10.1038/sj.onc.1203529

[14] Liu J , Qi Y , Li S , Hsu SC , Saadat S , Hsu J , et al. CREG1 interacts with Sec8 to promote cardiomyogenic differentiation and cell‐cell adhesion. Stem Cells 2016;34:2648–2660. 27334848 10.1002/stem.2434

[15] Liu Y , Tian X , Liu S , Liu D , Li Y , Liu M , et al. DNA hypermethylation: A novel mechanism of CREG gene suppression and atherosclerogenic endothelial dysfunction. Redox Biol 2020;32:101444. 32067910 10.1016/j.redox.2020.101444 PMC7264464

[16] Song HX , Tian X , Liu D , Liu M , Liu Y , Liu J , et al. CREG1 improves the capacity of the skeletal muscle response to exercise endurance via modulation of mitophagy. Autophagy 2021;17:4102–4118. 33726618 10.1080/15548627.2021.1904488 PMC8726698

[17] Guardiola O , Andolfi G , Tirone M , Iavarone F , Brunelli S , Minchiotti G . Induction of acute skeletal muscle regeneration by cardiotoxin injection. J Vis Exp 2017;119:54515. 10.3791/54515 PMC5407614 28117768

[18] Sévère N , Miraoui H , Marie PJ . The Casitas B lineage lymphoma (Cbl) mutant G306E enhances osteogenic differentiation in human mesenchymal stromal cells in part by decreased Cbl‐mediated platelet‐derived growth factor receptor alpha and fibroblast growth factor. J Biol Chem 2011;286:24443–24450. 21596750 10.1074/jbc.M110.197525 PMC3129223

[19] Brennan T , Adapala NS , Barbe MF , Yingling V , Sanjay A . Abrogation of Cbl‐PI3K interaction increases bone formation and osteoblast proliferation. Calcif Tissue Int 2011;89:396–410. 21952831 10.1007/s00223-011-9531-z PMC3191294

[20] Dieudonne FX , Severe N , Biosse‐Duplan M , Jingjie W , Yeu S , Pierre JM . Promotion of osteoblast differentiation in mesenchymal cells through Cbl‐mediated control of STAT5 activity. Stem Cells 2013;31:1340–1349. 23533197 10.1002/stem.1380

[21] Choi YH , Han Y , Lee SH , Jin YH , Bahn M , Hur KC , et al. Cbl‐b and c‐Cbl negatively regulate osteoblast differentiation by enhancing ubiquitination and degradation of Osterix. Bone 2015;75:201–209. 25744063 10.1016/j.bone.2015.02.026

[22] Fu X , Zhao JX , Liang J , Zhu MJ , Foretz M , Viollet B , et al. AMP‐activated protein kinase mediates myogenin expression and myogenesis via histone deacetylase 5. Am J Physiol Cell Physiol 2013;305:C887–C895. 23926128 10.1152/ajpcell.00124.2013 PMC3798682

[23] Fu X , Zhao JX , Zhu MJ , Foretz M , Viollet B , Dodson MV , et al. AMP‐activated protein kinase alpha1 but not alpha2 catalytic subunit potentiates myogenin expression and myogenesis. Mol Cell Biol 2013;33:4517–4525. 24043309 10.1128/MCB.01078-13 PMC3838187

[24] Yokouchi M , Kondo T , Houghton A , Bartkiewicz M , Horne WC , Zhang H , et al. Ligand‐induced ubiquitination of the epidermal growth factor receptor involves the interaction of the c‐Cbl RING finger and UbcH7. J Biol Chem 1999;274:31707–31712. 10531381 10.1074/jbc.274.44.31707

[25] Lyle C , Richards S , Yasuda K . c‐Cbl targets PD‐1 in immune cells for proteasomal degradation and modulates colorectal tumor growth. Sci Rep 2019;9:20257. 31882749 10.1038/s41598-019-56208-1 PMC6934810

[26] Weissman AM . Themes and variations on ubiquitylation. Nat Rev Mol Cell Biol 2001;2:169–178. 11265246 10.1038/35056563

[27] Deng M, Yang X , Qin B , Liu T , Zhang H , Guo W , et al. Deubiquitination and activation of AMPK by USP10. Mol Cell 2016;61:614–624. 26876938 10.1016/j.molcel.2016.01.010 PMC4836875

[28] Ghobrial G , Araujo L , Jinwala F , Li S , Lee LY . The structure and biological function of CREG. Front Cell Dev Biol 2018;6:136. 30416997 10.3389/fcell.2018.00136 PMC6212480

[29] Shen M , Yen A . c‐Cbl interacts with CD38 and promotes retinoic acid‐induced differentiation and G0 arrest of human myeloblastic leukemia cells. Cancer Res 2008;68:8761–8769. 18974118 10.1158/0008-5472.CAN-08-1058 PMC4896297

[30] Molero JC , Jensen TE , Withers PC , Couzens M , Herzog H , Thien CBF , et al. c‐Cbl‐deficient mice have reduced adiposity, higher energy expenditure, and improved peripheral insulin action. J Clin Invest 2004;114:1326–1333. 15520865 10.1172/JCI21480 PMC524227

[31] Azakir BA , Angers A . Reciprocal regulation of the ubiquitin ligase itch and the epidermal growth factor receptor signaling. Cell Signal 2009;21:1326–1336. 19341794 10.1016/j.cellsig.2009.03.020

[32] Zuo W , Huang F , Chiang YJ , Li M , du J , Ding Y , et al. c‐Cbl‐mediated neddylation antagonizes ubiquitination and degradation of the TGF‐β type II receptor. Mol Cell 2013;49:499–510. 23290524 10.1016/j.molcel.2012.12.002

[33] Lyle CL , Belghasem M , Chitalia VC . c‐Cbl: An important regulator and a target in angiogenesis and tumorigenesis. Cell 2019;8:498. 10.3390/cells8050498 PMC6563115 31126146

[34] Višnjić D , Lalić H , Dembitz V , Tomić B , Smoljo T . AICAr, a widely used AMPK activator with important AMPK‐independent effects: a systematic review. Cell 2021;10:1095. 10.3390/cells10051095 PMC8147799 34064363

[35] Pineda CT , Potts PR . Oncogenic MAGEA‐TRIM28 ubiquitin ligase downregulates autophagy by ubiquitinating and degrading AMPK in cancer. Autophagy 2015;11:844–846. 25945414 10.1080/15548627.2015.1034420 PMC4509443

[36] Panci G , Chazaud B . Inflammation during post‐injury skeletal muscle regeneration. Semin Cell Dev Biol 2021;119:32–38. 34140216 10.1016/j.semcdb.2021.05.031

[37] Graca FA , Stephan A , Minden‐Birkenmaier BA , Shirinifard A , Wang YD , Demontis F , et al. Platelet‐derived chemokines promote skeletal muscle regeneration by guiding neutrophil recruitment to injured muscles. Nat Commun 2023;14:2900. 37217480 10.1038/s41467-023-38624-0 PMC10203137

[38] Yin J , Yang L , Xie Y , Liu Y , Li S , Yang W , et al. Dkk3 dependent transcriptional regulation controls age related skeletal muscle atrophy. Nat Commun 2018;9:1752. 29717119 10.1038/s41467-018-04038-6 PMC5931527

[39] de Wilde J , Hulshof MF , Boekschoten MV , de Groot P , Smit E , Mariman ECM . The embryonic genes Dkk3, Hoxd8, Hoxd9 and Tbx1 identify muscle types in a diet‐independent and fiber‐type unrelated way. BMC Genomics 2010;11:176. 20230627 10.1186/1471-2164-11-176 PMC2847971

[40] von Haehling S , Coats AJS , Anker SD . Ethical guidelines for publishing in the *Journal of Cachexia, Sarcopenia and Muscle*: update 2021. J Cachexia Sarcopenia Muscle 2021;12:2259–2261. 34904399 10.1002/jcsm.12899 PMC8718061

