# Supplementary material for: CREG1 deficiency impaired myoblast differentiation and skeletal muscle regeneration
Source: J Cachexia Sarcopenia Muscle. 2024 Jan 25;15(2):587–602. doi: 10.1002/jcsm.13427 (PMC10995283; doi:10.1002/jcsm.13427)
Supplement: Supplementary file 1 — Figure S1. CREG1 expression was associated with skeletal muscle regeneration. (A‐B) Volcano plot; Heatmap; Quantitation of upregulated and downregulated transcriptional profiles of the skeletal muscle from healthy older (N = 25) and younger (N = 26) adult men and women in GSE8479 database. (a: older people before exercise; b: older people after exercise) (C) Real‐time PCR and western blot analysis detected expression of CREG1 in C2C12 cells differentiation from 1 to 4 days. (n = 3). (D) Schematic of CTX injury model. (E) Real time PCR analysis of Creg1, Pax7, Myod1, MyoG and Myf5 expression in the TA muscles. (n = 3). (F) Western blot analysis revealed proteins expression in the TA muscles (n = 3). For all statistical plots, data are shown as mean ± SEM, **p < 0.01, #p < 0.05. dpi: days post‐injury; CREG1: cellular repressor of E1A‐stimulated genes 1; TA: tibialis anterior. Figure S2. Generation of Creg1 knockdown mice by adeno‐associated virus serotype 9 (AAV9). (A) A chart described an organization knockdown strategy. (B) Realtime PCR detected Creg1 mRNA expression in tibialis anterior (TA) and Gastrocnemius (GAS). (n = 3). (C‐D) Western blot showed the expression of CREG1 in tibialis anterior (TA) and Gastrocnemius (GAS). (n = 3). (E) Representative H&E and immunofluorescent staining analysis of TA muscles in cross‐sectional area (CSA), (n = 5). scale bars: 50 μm. (F) Representative H&E staining examined regeneration of TA muscle at 3 dpi, EMH + muscle fibres detected by IHC staining in TA muscle at 3 dpi, scale bars: 50 μm. n = 5. For all statistical plots, data are shown as mean ± SEM, **p < 0.01. Statistical significance was determined by Student‘s t test. Dpi: days post‐injury; CREG1: cellular repressor of E1Astimulated genes 1; TA: tibialis anterior; GAS: Gastrocnemius; CSA: cross‐sectional area. Figure S3. CREG1 silence and overexpression in C2C12 cells. (A‐C) Western blot analysis showed expression of CREG1 in loss‐ and gain‐of function approach. For all s [file JCSM-15-587-s001.pdf]

# Supplemental materials

# MATERIALS AND METHODS

***Western blot and antibodies.***

Cells and homogenized TA tissues were lysed in ice-cold RIPA buffer (Thermo Fisher Scientific, UJ289235) for 30 minutes and then centrifuged at 12,000 × g for 10 min at 4° C. Equal amounts of samples were separated by SDS-PAGE (at 120 V for 1 h), and then proteins were transferred onto a polyvinylidene difluoride membrane (Merck Millipore Ltd. R9KA84149). Membranes were blocked in 5% nonfat milk, then were incubated with antibodies against CREG1 (HUABIO, ER61836), GAPDH (Cell Signaling Technology, 5174S), MYOD1 (Cell Signaling Technology, 13812S), MYOG (Abcam, ab1385), MYF5 (Abcam, ab125301), PAX7 (Sigma, QC11932), MyHC (DSHB, MF20C), C-CBL (Cell Signaling Technology, 2747S), LKB1 (Cell Signaling Technology, 3047S), CAMKK2 (HUABIO, BJ05125116), AMPKa (Cell Signaling Technology, 2532S), p-AMPKa (Cell Signaling Technology, 2531S), AMPKa1 (Abcam, ab3759), AMPKa2 (Cell Signaling Technology, 2757S), GFP (Abcam, ab290), FLAG (Sigma, F1840), HA (Abcam, ab1424), DKK3 (MCE, TA349296S), ubiquitin (Cell Signaling Technology, 3936) at 4°C overnight, all antibodies were diluted 1:1000. After incubation, three washes with TBST, membranes were incubated the HRP-conjugated secondary antibody (1:5000; Jackon ImmunoResearch, 150783) for 2 h at room temperature. After four washes with TBST, membranes were scanned with ECL in Amersham Imager680 (Tokyo, Japan).

***Plasmids, adenoviral vectors construction and siRNA***

AMPKa1 full-length and fragments: AMPKa1 pcDNA3.1-GFP,1-312 pcDNA3.1-GFP, 312-392 pcDNA3.1-GFP, 392-558 pcDNA3.1-GFP; C-CBL pLVX-mCherry-N1 and pcDNA3.1-CMV-Itch-GFP were obtained from GENEWIZ (China). The series of GFP-tagged AMPKa1 mutants: K71R, K265R, K396R and K485R (the lysine at positions 71, 265, 396 and 485 substituted by arginine); Ubiquitin mutants: K48R and K63R (the lysine at positions 48 and 63 substituted by arginine); myc-tagged C-CBL mutants (C-CBL-70Z); pcDNA3.1-CMV-Creg1-3flag, pcDNA3.1-ubiquitin-HA and control, above all of these were generated from WZ Biosciences Inc. Adenoviral vector, including Ad-GFP, AdC-CBL-3flag and AdCREG1-3flag (OBiO Technology Corp). pcDNA3.1-GFP control, mC-CBL 1-359 pLVX-mCherry, mC-CBL 359-912 pLVX-mCherry-N1 were obtained from *WZ Biosciences* (China). siCreg1 RNA (RIBOBIO, China) targeting sequence: #1:GCCACTATCTCCACAATAA, #2:CTACTTTGGTGGACCTAAA, #3:GCTGACTATGTCTTTAGCA. siC-Cbl RNA (RIBOBIO, China) targeting sequence: #1:CTCGGAGAATCAACTCAGA, #2:CAATGAGGGTTCCCAAGTT, #3:GCTCTCAAGTGAGATTGAA. siAmpka1 RNA (RIBOBIO, China) targeting sequence: #1:GCAGAAGATTCGGAGCCTT, #2:GCACACCCTGGATGAATTA, #3:GCAGAAGTTTGTAGAGCAA.

***Real-time PCR.***

As described previously,[16] according to the Eastep® Super kit's instructions (Promega, 0000287896), RNA was reverse transcribed using the SuperScript™ III First-Strand Kit (Thermo Fisher Scientific, 18080400). Real-time PCR was performed on an ABI 7300 PCR System. Primers are listed in supplemental materials.

*Creg1*-F: 5'-TGTCGGGAACTGTGACCAAG-3';

*Creg1*-R:5'-CTTTAGTTGTTGAAATCTGTG-3';

*Gapdh*-F: 5'-TCAACGACCCCTTCATTGAC-3';

*Gapdh*-R: 5'-ATGCAGGGATGATGTTCTGG-3';

*Myh1*-F: 5'-ACTGTCAACACTAAGAGGGTCA-3';

*Myh1*-R:5'-GGCAG TTGGATGATTTGATCTTCCAGGG' -3;

*Myh2/Mhc2a*-F: 5'-AAGTGACTGTGAAAACAGAAGCA-3'

*Myh2/Mhc2a*-R: 5'-GCAGCCATTTGTAAGGGTTGAC-3';

*Mhc2b*-F: 5'-TTGAAAAGACGAAGCAGCGAC-3'

*Mhc2b*-R:5'-AGAGAGCGGGACTCCTTCTG-3'.

*C-Cbl*-F:5'-CATTGCCAATACCTCCCACAC-3';

*C-Cbl* -R: 5'-TTTAGGGATGGTCCGAGACAA-3'

*Lkb1*-F:5'-GAGGACGGCATTATCTACACCCA-3';

*Lkb1*-R: 5'-GCTCTGTCCATTCTGACCCACTT -3';

*Camkk2*-F:5'-CCAGGATTGTGGTGCCTGAAATC-3';

*Camkk2*-R: 5'-ATTCTCGACCTCCTCTTCGGTCA -3';

*Pax7*-F:5'-TTGGGGAACACTCCGCTGTGC-3';

*Pax7*-R: 5'-CAGGGCTTGGGAAGGGTTGGC-3';

*Myod1*-F:5'-TCTGGAGCCCTCCTGGCACC-3';

*Myod1*-R: 5'-CGGGAAGGGGGAGAGTGGGG -3';

*Myf5*-F:5'-AAACTCCGGGAGCTCCGCCT-3';

*Myf5*-R: 5'-GGCAGCCGTCCGTCATGTCC-3';

*MyoG*-F:5'-GAGATCCTGCGCAGCGCCAT -3';

*MyoG*-R: 5'-CCCCGCCTCTGTAGCGGAGA -3';

*Ampka1*-F:5'-GTCAAAGCCGACCCAATGATA -3';

*Ampka1*-R: 5'-CGTACACGCAAATAATAGGGGTT -3';

*Ampka2*-F:5'-CAGGCCATAAAGTGGCAGTTA -3';

*Ampka2*-R: 5'-AAAAGTCTGTCGGAGTGCTGA -3';

*Itch*-F:5'-TGGGTAGTCTGACCATGAAATCT-3';

*Itch*-R: 5'-GGGGTAACAATAACTGTGAGGG-3';

*IL-6*-F:5’-CCAAGAGGTGAGTGCTTCCC -3’;

*IL-6*-R: 5’-CTGTTGTTCAGACTCTCTCCCT -3’;

*IL-1beta*-F:5’-GCAACTGTTCCTGAACTCAACT-3’;

*IL-1beta*-R: 5’-ATCTTTTGGGGTCCGTCAACT-3’;

*TNFa*-F:5’-GACGTGGAACTGGCAGAAGAG-3’;

*TNFa*-R: 5’-TTGGTGGTTTGTGAGTGTGAG-3’;

*Dkk3*-F:5’-CTCGGGGGTATTTTGCTGTGT-3’;

*Dkk3*-R: 5’-TCCTCCTGAGGGTAGTTGAGA-3’;

*18S*-F:5’-TGCTGTCCCTGTATGCCTCT-3’;

*18S*-R: 5’-TGTAGCCACGCTCGGTCA-3’;

***Mass spectrometry (MS).***

As described previously,[1] Mass spectrometry analyses were performed in an LC–MS/MS system (Ekspert™ nanoLC, ABSciex Triple TOF™ 5600-plus), and the data analysis was using MaxQuant 1.5.5.1.

***RNA-Seq transcriptomic assay***

Total RNA was quantified using a NanoDrop ND-2000 (Thermo Scientific), and RNA integrity was assessed using an Agilent Bioanalyzer 2100 (Agilent Technologies). Sample labeling, microarray hybridization, and washing were performed according to the manufacturer’s standard protocols. Briefly, total RNA was transcribed to double-stranded cDNA, then synthesized into cRNA and labeled with Cyanine-3-CTP. The labeled cRNAs were hybridized to the microarray. After washing, the arrays were scanned using an Agilent Scanner G2505C (Agilent Technologies).

***Cell differentiation index Assays and Creatine kinase (CK) activity assays.***

Cell differentiation index Assays and quantification were carried out according to manufacturer’s instructions. CK activity was measured using a Creatine Kinase Activity AssayKit (Sigma) according to the manufacturer's instructions.

***Exercise tolerance.***

All mice were adapted to the run-to-exhaustion protocol on a motorized treadmill, as previous study described [16].

## Supplemental Figure 1

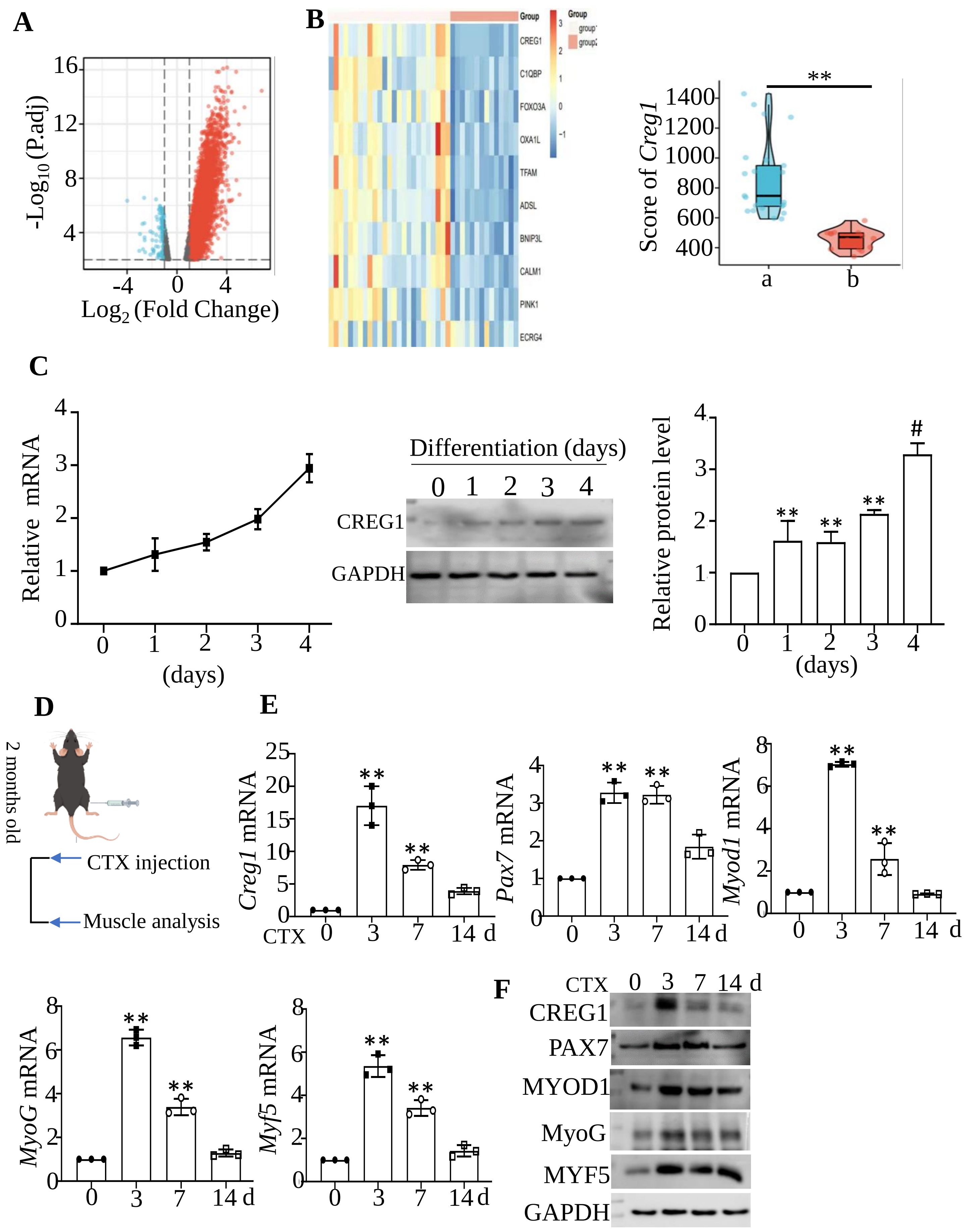

**Supplemental Figure 1.** CREG1 expression was associated with skeletal muscle regeneration. (**A-B**) Volcano plot; Heatmap; Quantitation of upregulated and downregulated transcriptional profiles of the skeletal muscle from healthy older (N = 25) and younger (N = 26) adult men and women in GSE8479 database. (a: older people before exercise; b: older people after exercise) (**C**) Real-time PCR and western blot analysis detected expression of CREG1 in C2C12 cells differentiation from 1 to 4 days. (n=3). (**D**) Schematic of CTX injury model. (**E**) Real time PCR analysis of *Creg1*, *Pax7*, *Myod1*, *MyoG* and *Myf5* expression in the TA muscles. (n=3). (**F**) Western blot analysis revealed proteins expression in the TA muscles (n=3). For all statistical plots, data are shown as mean ± SEM, $^{**}p < 0.01$, $^{\#}p < 0.05$. dpi: days post-injury; CREG1: cellular repressor of E1A-stimulated genes 1; TA: tibialis anterior.

# Supplemental figure 2

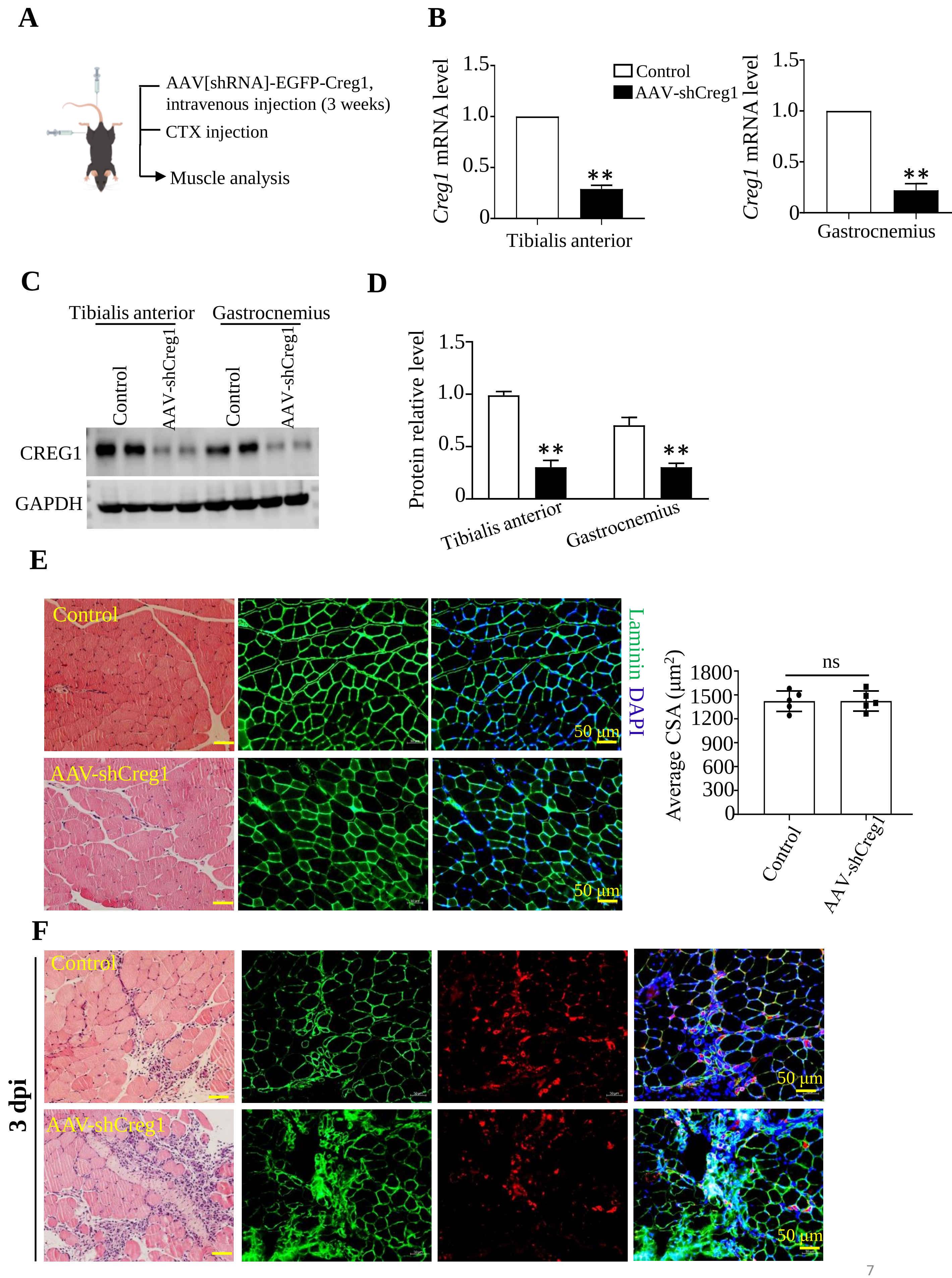

**Supplemental Figure 2.** Generation of Creg1 knockdown mice by adeno-associated virus serotype 9 (AAV9). (**A**) A chart described an organization knockdown strategy. (**B**) Real-time PCR detected *Creg1* mRNA expression in tibialis anterior (TA) and Gastrocnemius (GAS). (n=3). (**C-D**) Western blot showed the expression of CREG1 in tibialis anterior (TA) and Gastrocnemius (GAS). (n=3). (**E**) Representative H&E and immunofluorescent staining analysis of TA muscles in cross-sectional area (CSA), (n=5). scale bars: 50 μm. (**F**) Representative H&E staining examined regeneration of TA muscle at 3 dpi, $EMH^+$ muscle fibers detected by IHC staining in TA muscle at 3 dpi, scale bars: 50 μm. n=5. For all statistical plots, data are shown as mean ± SEM, $^{**}p < 0.01$. Statistical significance was determined by Student's *t* test. dpi: days post-injury; CREG1: cellular repressor of E1A-stimulated genes 1; TA: tibialis anterior; GAS : Gastrocnemius ; CSA: cross-sectional area.

# Supplemental figure 3

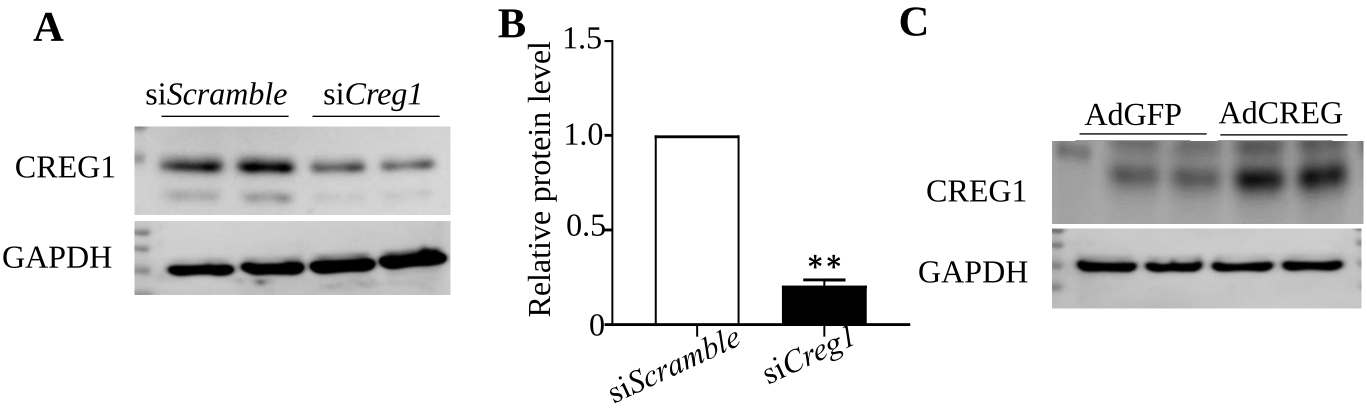


**Supplemental Figure 3.** CREG1 silence and overexpression in C2C12 cells. (**A-C**) Western blot analysis showed expression of CREG1 in loss- and gain-of function approach. For all statistical plots, data are shown as mean ± SEM, $^{**}p < 0.01$. Statistical significance was determined by Student's *t* test.

# Supplemental Figure 4

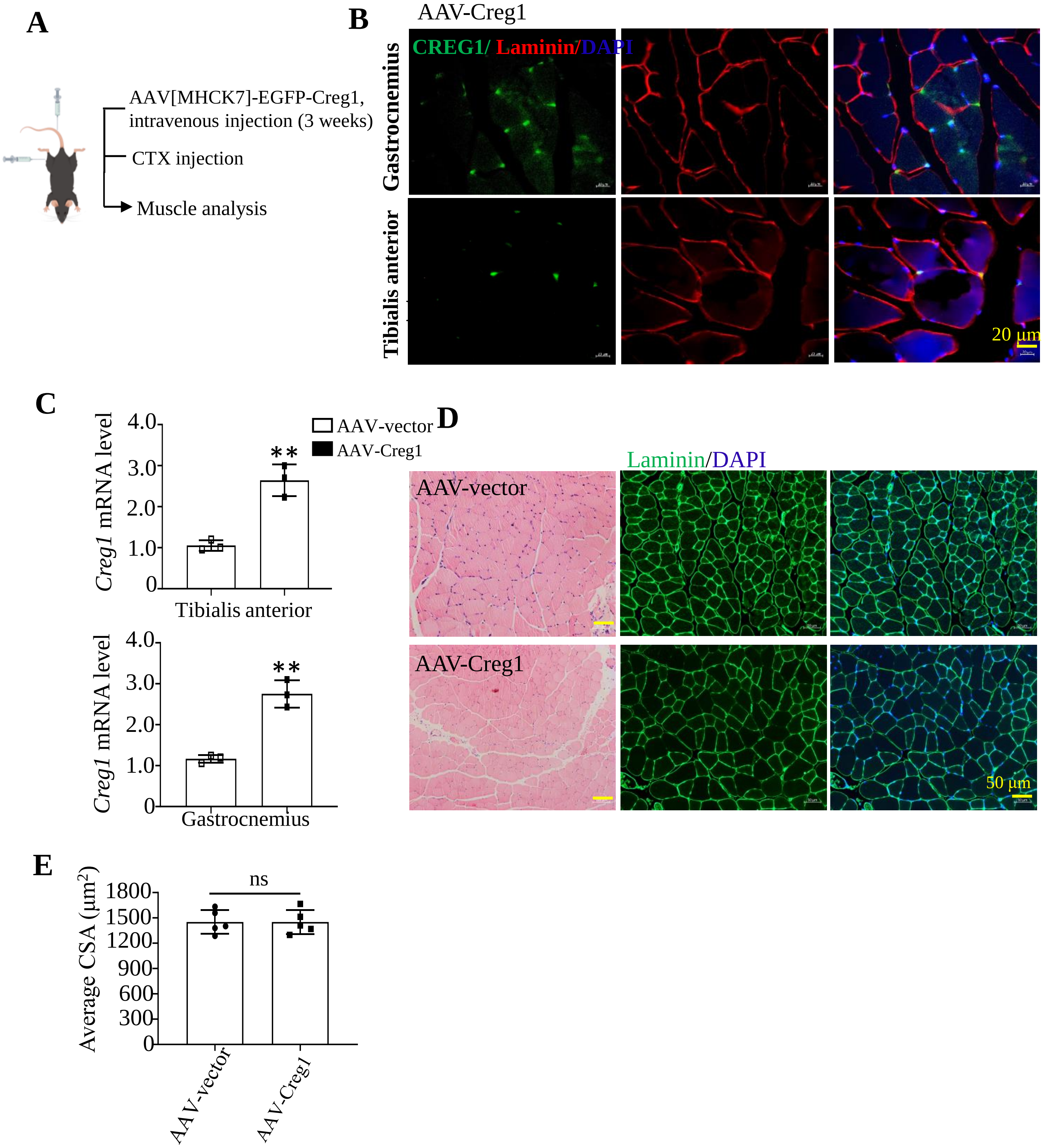

**Supplemental Figure 4.** Generation of muscle satellite cells specific Creg1 over-expression mice by adeno-associated virus serotype 9 (AAV9). (**A**) A chart described an organization overexpression strategy. (**B**) Immunofluorescent staining analysis examined the expression of CREG1 and Laminin, scale bars: 20 μm. n=5.(**C**) Real-time PCR detected *Creg1* mRNA expression in tibialis anterior (TA) and Gastrocnemius (GAS). n=3. (**D-E**) Representative H&E and immunofluorescent staining analysis of TA muscles in cross-sectional area (CSA), scale bars: 50 μm. n=5. For all statistical plots, data are shown as mean ± SEM, $^{**}p < 0.01$. Statistical significance was determined by Student's *t* test. CREG1: cellular repressor of E1A-stimulated genes 1; TA: tibialis anterior; GAS: Gastrocnemius; CSA: cross-sectional area.

# Supplemental figure 5

**A**

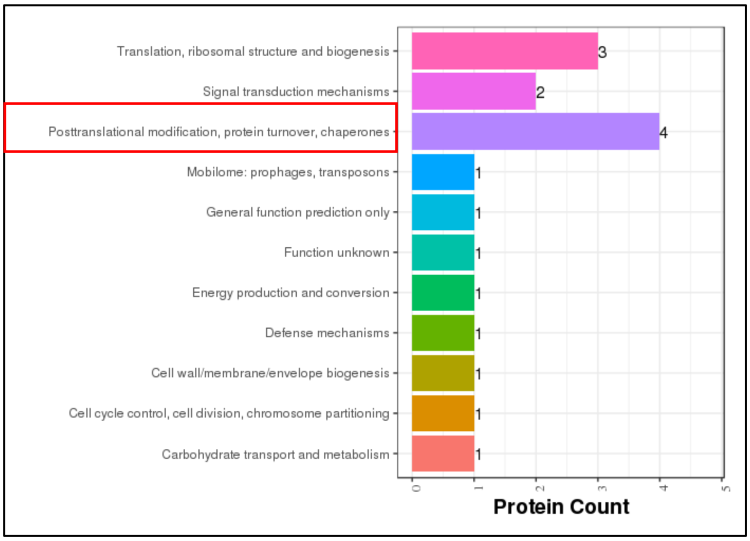


**B**

| Protein | Full name | Related signaling pathway |
|---|---|---|
| **CBL** | Casitas b-lineage lymphoma | Endocytosis; **Insulin signaling pathway**; T cell receptor signaling pathway; ErbB signaling pathway; **Ubiquitin mediated proteolysis** |
| CDC42 | Cell division control protein 42 homolog | Endocytosis; Regulation of actin cytoskeleton; MAPK signaling pathway; Leukocyte transendothelial migration; T cell receptor signaling pathway; Chemokine signaling pathway; VEGF signaling pathway; Ras signaling pathway; RAGE signaling pathway in diabetic complications |
| DDX5 | DEAD box protein 5 | Spliceosome; Proteoglycans in cancer; Transcriptional misregulation in cancer |
| CAPZB | F-actin-capping protein subunit beta | Endocytosis |
| ARPC2 | Actin-related protein complex subunit 2 | Endocytosis, Regulation of actin cytoskeleton |

**C**

Integrated Proteomics: protein expression in normal tissues and cell lines from ProteomicsDB, MaxQB, and MOPED for CBL Gene

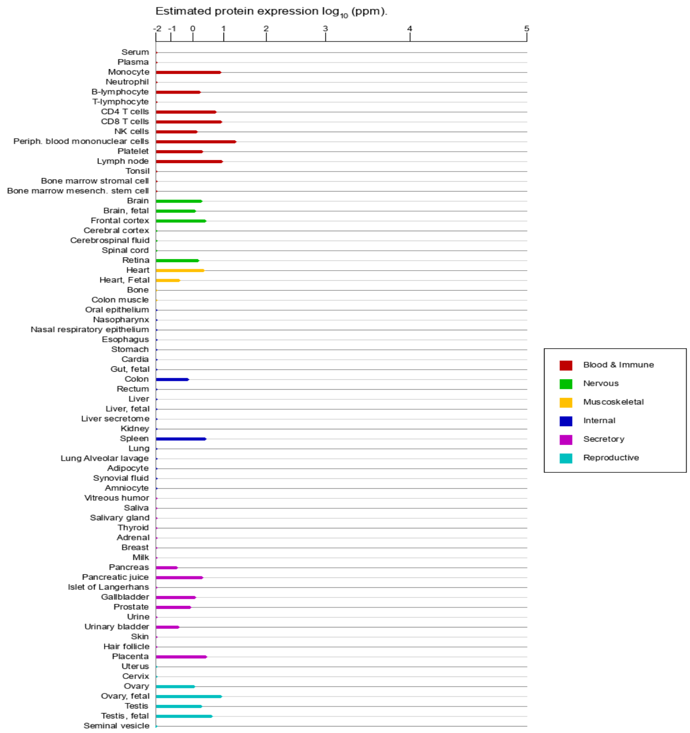




**Supplemental Figure 5.** Mass Spectrometric analysis.

## Supplemental figure 6

A

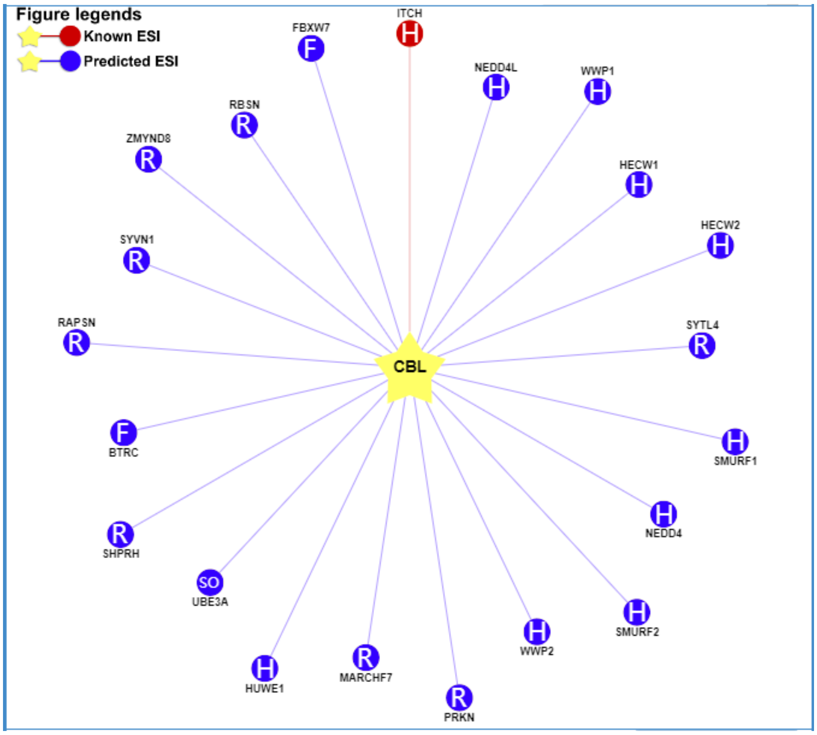


B

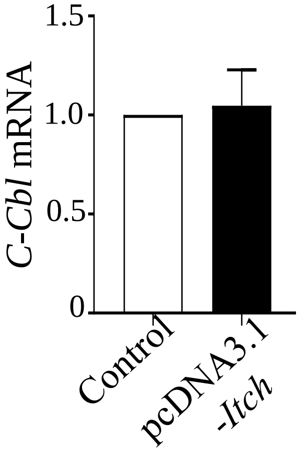


**Supplemental Figure 6.** ITCH interacted with C-CBL in C2C12 cells. (**A**) Some E3 ubiquitin ligases were predicted in UbiBrowser 2.0 database (http://ubibrowser. bio-it. cn/ubibrowser_v3/). (**B**) RT-PCR analysis of *C-Cbl* expression, n=3. For all statistical plots, data are shown as mean ± SEM. Statistical significance was determined by Student's *t* test.

# Supplemental figure 7

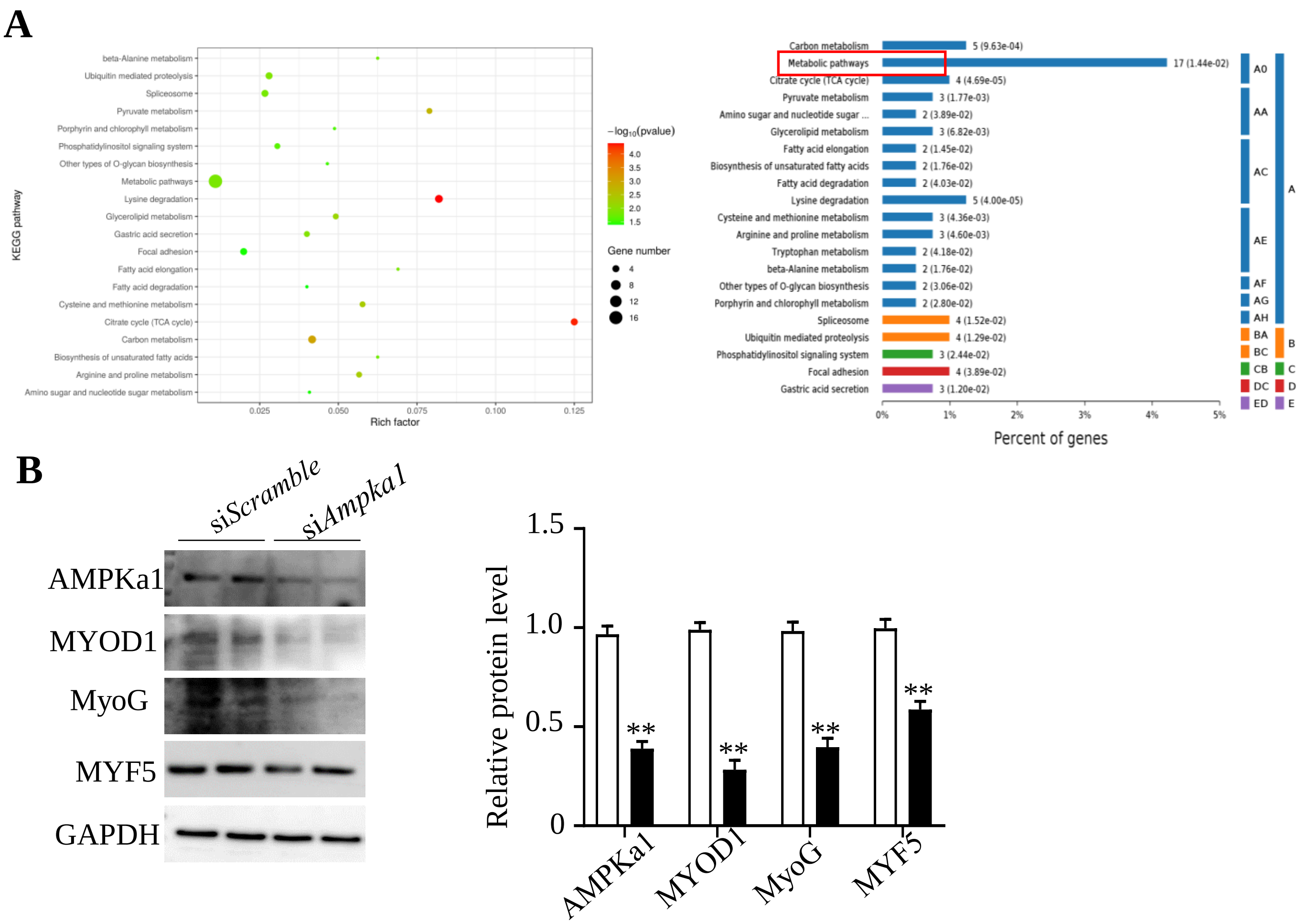


**Supplemental Figure 7.** C-CBL interacted with AMPKa1 in C2C12 cells. (**A**) Mass Spectrometric analysis. (**B**) Western blot analysis showed expression of AMPKa1, MYOD1, MyoG and MYF5. n=3. For all statistical plots, data are shown as mean ± SEM, $**p < 0.01$. Statistical significance was determined by Student's $t$ test.

# Supplemental figure 8

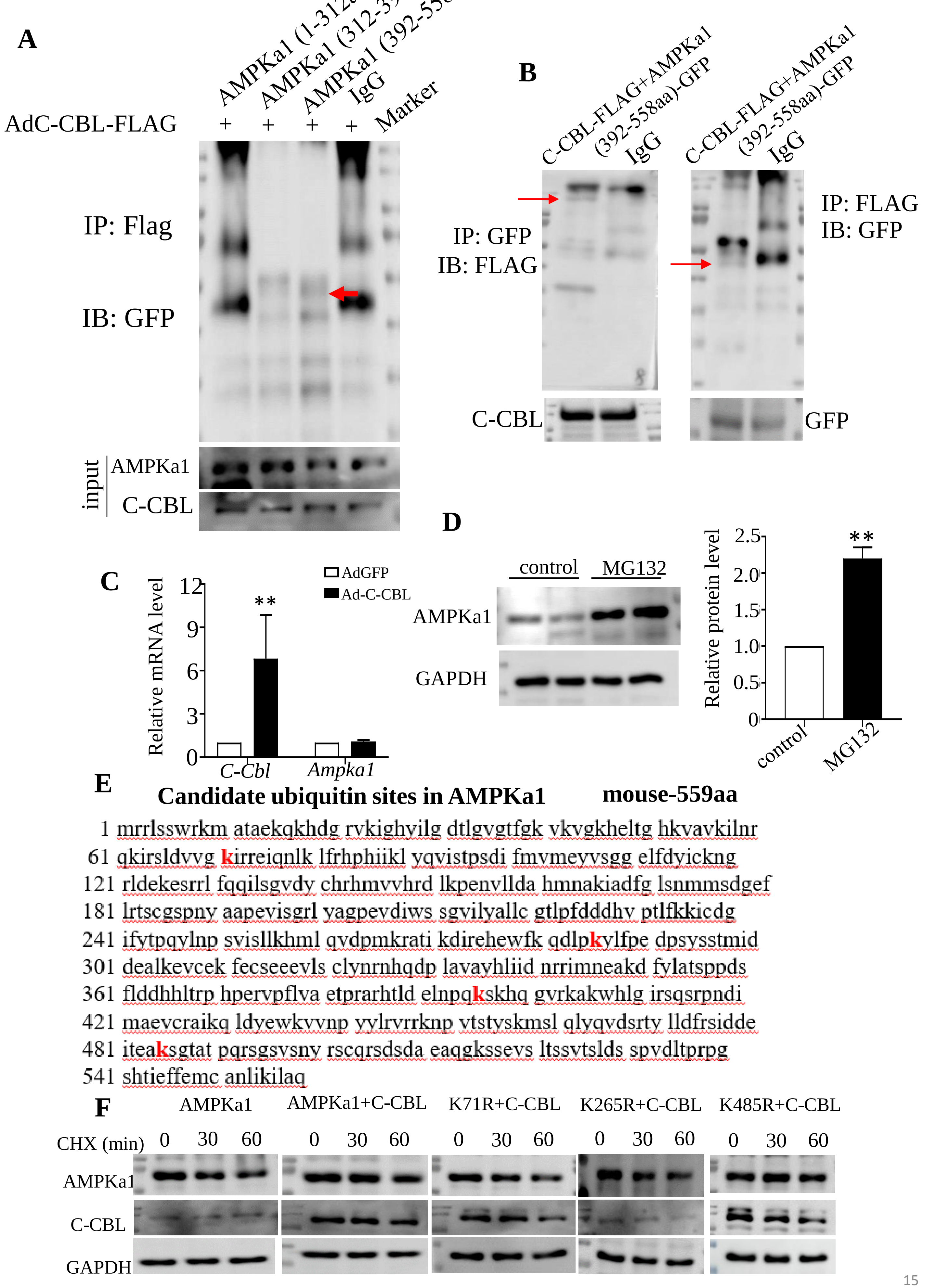

**Supplemental Figure 8.** C-CBL is an E3 Ligase of AMPKa1, related to Figure 6. (**A-B**) The interaction between C-CBL-Flag and AMPKa1-GFP in HEK293T cells was evaluated by co-immunoprecipitation (Co-IP). (**C**) RT-PCR analysis of *C-Cbl* and *Ampka1* expression, n=3. (**D**) Western blot analysis showed expression of AMPKa1 with or without MG132 administrated. (**E**) Candidate ubiquitin sites in AMPKa1. (**F**) Representative western blot showing the protein expression of wild type, K71R, K265R or K485R mutants of AMPKa1 in HEK293T cells at the indicated time points after Cycloheximide (CHX, 20 μg/mL) treatment. n=3, data are shown as mean ± SEM, $^{**}p < 0.01$. Statistical significance was determined by Student's *t* test.

## Supplemental figure 9

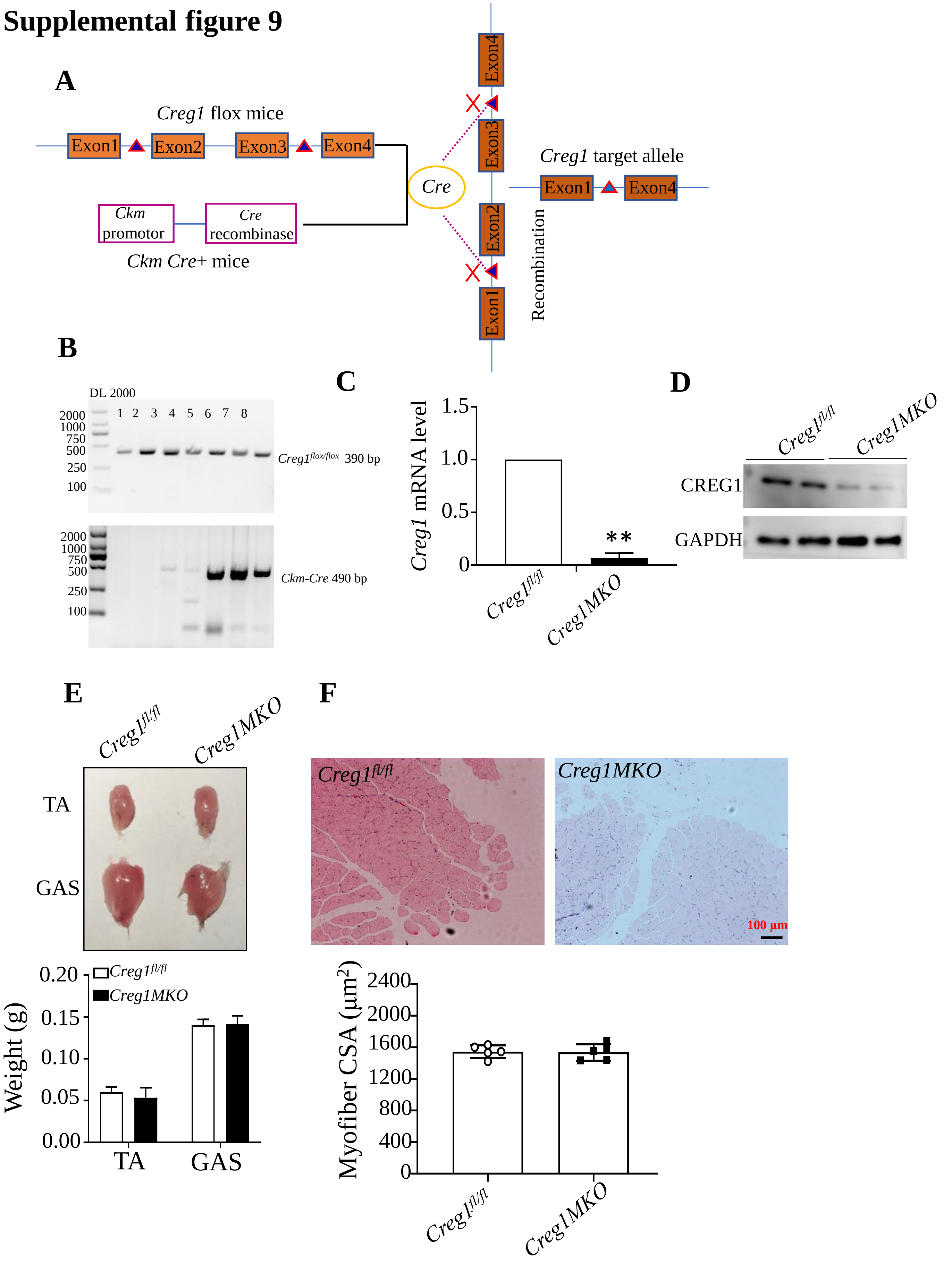

**Supplemental Figure 9.** Generation of creg1 skeletal muscle-specific knockout mice. (**A**) A chart described an organization-specific knockout strategy. (**B**) Real-time PCR detected *Creg1* gene and *Cre* gene expression. (**C-D**) Real-time PCR and western blot showed the expression of CREG1 in *tibialis anterior* (TA) of $Creg1^{fl/fl}$ and *Creg1MKO*. n=3. (**E**) A TA and gastrocnemius (GAS) muscles representative image and quantification from $Creg1^{fl/fl}$ and *Creg1MKO* mice. (**F**) H&E staining examined TA muscle at uninjury, scale bars: 100 μm. Average values of myofiber cross-sectional areas (CSA). n=5. For all statistical plots, data are shown as mean ± SEM, *$p$ < 0.05, **$p$ < 0.01. Statistical significance was determined by Student's *t* test. TA: tibialis anterior; GAS: Gastrocnemius; CSA: cross-sectional area.

# Supplemental figure 10

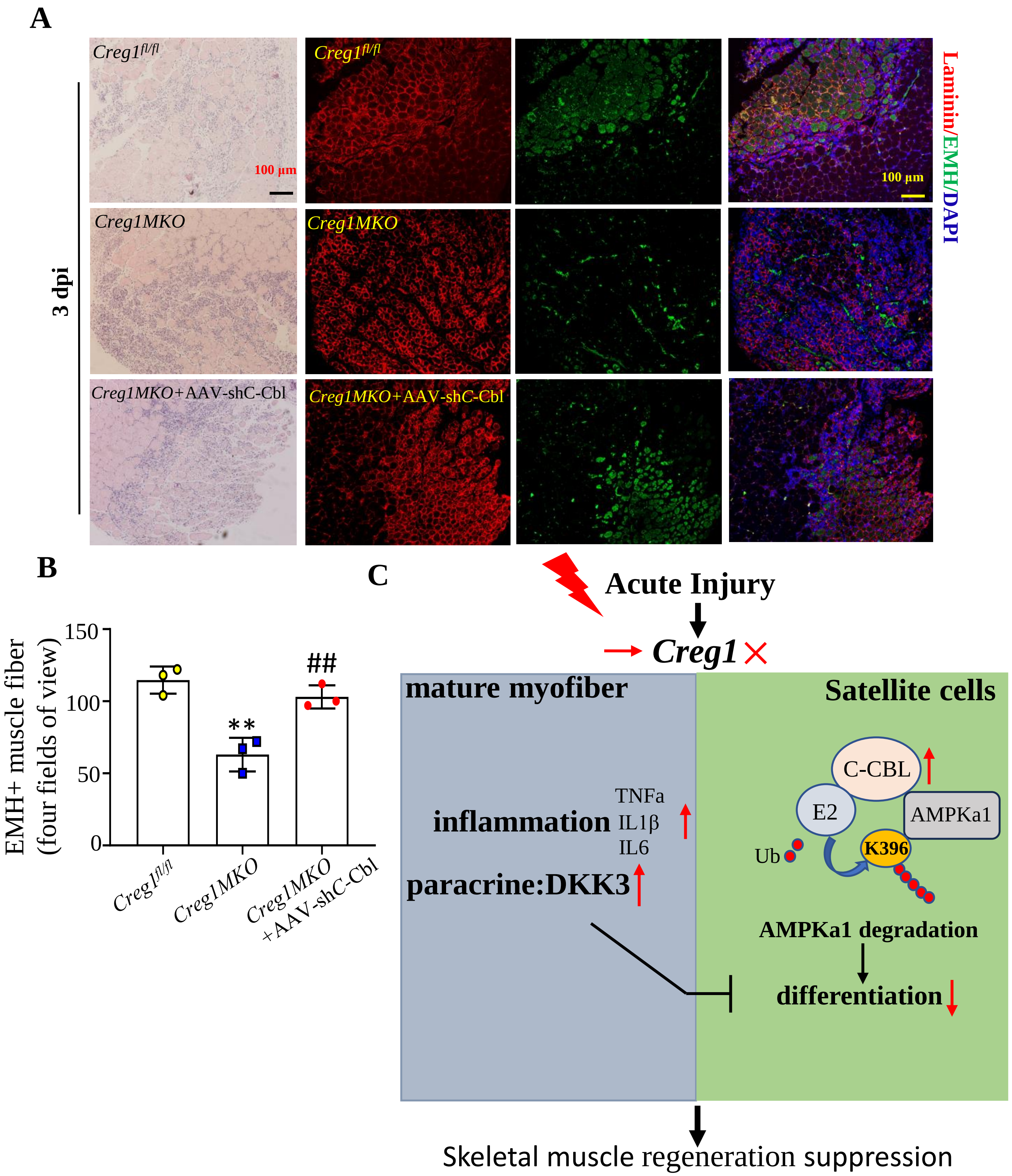


**Supplemental Figure 10.** C-CBL knockdown in TA muscle of *Creg1MKO* mice improved muscle regeneration. (**A-B**) Representative H&E staining examined regeneration of TA muscle at 3 dpi, scale bars: 100 μm. $EMH^{+}$ muscle fibers detected by IHC staining in TA muscle at 3 dpi and quantification, scale bars: 100 μm. n=3. (C) A schematic picture. For all statistical plots, data are shown as mean ± SEM, $^{**}p < 0.01$, $^{\#\#}p < 0.01$. TA: tibialis anterior.
